# Designer Receptors for Nucleotide‐Resolution Analysis of Genomic 5‐Methylcytosine by Cellular Imaging

**DOI:** 10.1002/anie.202001935

**Published:** 2020-04-07

**Authors:** Álvaro Muñoz‐López, Benjamin Buchmuller, Jan Wolffgramm, Anne Jung, Michelle Hussong, Julian Kanne, Michal R. Schweiger, Daniel Summerer

**Affiliations:** ^1^ Faculty of Chemistry and Chemical Biology TU Dortmund University Otto-Hahn Str. 6 44227 Dortmund Germany; ^2^ International Max Planck Research School Max Planck Institute of Molecular Physiology Otto-Hahn Str. 10 44227 Dortmund Germany; ^3^ Department of Epigenetics and Tumor Biology, Medical Faculty University of Cologne Kerpener Str. 62 50937 Köln Germany

**Keywords:** biosensors, DNA methylation, epigenetics, imaging probes, membrane-less organelles

## Abstract

We report programmable receptors for the imaging‐based analysis of 5‐methylcytosine (5mC) in user‐defined DNA sequences of single cells. Using fluorescent transcription‐activator‐like effectors (TALEs) that can recognize sequences of canonical and epigenetic nucleobases through selective repeats, we imaged cellular SATIII DNA, the origin of nuclear stress bodies (nSB). We achieve high nucleobase selectivity of natural repeats in imaging and demonstrate universal nucleobase binding by an engineered repeat. We use TALE pairs differing in only one such repeat in co‐stains to detect 5mC in SATIII sequences with nucleotide resolution independently of differences in target accessibility. Further, we directly correlate the presence of heat shock factor 1 with 5mC at its recognition sequence, revealing a potential function of 5mC in its recruitment as initial step of nSB formation. This opens a new avenue for studying 5mC functions in chromatin regulation in situ with nucleotide, locus, and cell resolution.

## Graphical abstract

**It needs two TALEs to tango**: Programmable receptors for the imaging‐based analysis of 5‐methylcytosine (5mC) in DNA sequences of single cells are reported. Staining of SATIII DNA with pairs of 5mC‐sensitive and 5mC‐insensitive transcription‐activator‐like effectors (TALEs) allows the analysis of 5mC at a target nucleotide position and its direct correlation with the recruitment of transcription factor HSF1 as the initial step of nuclear stress body formation.

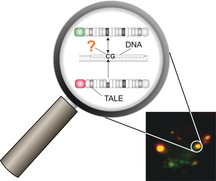

The epigenetic nucleobase 5‐methylcytosine (5mC, Figure 1 a) regulates transcription, cell differentiation, and development in mammalian genomes.^1^ 5mC is introduced into CpG dinucleotides by DNA methyltransferases (DNMT), and aberrant methylation is an early event in carcinogenesis.^2^ The main strategy to find clues to 5mC functions is its mapping in purified genomic DNA with nucleotide and strand resolution through bisulfite sequencing, and the correlation of identified 5mC sites with maps of other chromatin features.^3^ In contrast, methods for the imaging‐based in situ analysis of cellular 5mC with nucleotide and strand resolution have not yet been reported. These could enable direct observation of 5mC at user‐defined genomic positions of single cells, and their direct correlation with other imageable chromatin features.^4^

**Figure 1:**
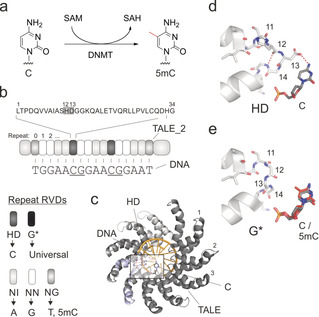
DNA recognition of TALEs. a) Cytosine 5‐methylation. SAM=S‐adenosylmethionine, SAH=S‐adenosylhomocysteine. b) TALE features. Repeat sequence on top with RVD in box. RVD selectivities below. c) Crystal structure of DNA‐bound TALE.^10^ Frame marks Figure 1 d. d) RVD HD bound to C. e) Model of RVD G* bound to C or 5mC.^11^

Cellular 5mC has been imaged using generic receptors like antibodies or methyl‐CpG‐binding domains^5^ in co‐stains with fluorescence in situ hybridization (FISH) probes,^6^ or with DNA‐binding proteins in fluorescence complementation designs^7^ to add locus information. However, employing two different receptor molecules with no or poorly defined connection does not offer nucleotide resolution analysis. FISH probes equipped with long chelator linkers for OsO_4_‐mediated crosslinking of 5mC offer more potential in this direction,^8^ but require harsh, oxidative staining conditions, and nucleotide/strand resolution has not been demonstrated.^9^

We aimed at developing purely recognition‐based imaging receptors that integrate sequence‐ and 5mC selectivity within one programmable scaffold. For engineering, we chose transcription‐activator‐like effector (TALE) proteins^12^ that bind one strand of duplex DNA through a modular domain of repeats, each recognizing one nucleobase through a repeat variable di‐residue (RVD, Figure 1 b–e).^13^ Selective repeats for epigenetic nucleobases are available,^11^, ^14^ enabling their analysis in purified genomic DNA with nucleotide and strand resolution.14e, 15 Moreover, TALEs have been used for cell imaging^16^ and in a mouse centromere example, imaging patterns indicated single‐nucleotide polymorphism selectivity.16a

We chose to target pericentromeric SATIII DNA, since this class of clustered repeats is the origin of nuclear stress bodies (nSB),^17^ a type of membrane‐less organelle exhibiting aberrant methylation in several cancers.^18^ The abundance of redundant SATIII sequences throughout the genome complicates their selective amplification, sequencing, and alignment, so that the genomic distribution and individual methylation of SATIII loci is poorly understood.^19^ In contrast, TALE‐based imaging could enable studying roles of SATIII methylation in nSB formation with cell and locus resolution.

To exert maximum control over the staining procedure and allow for potential applications in fixed tissue samples, for example, of clinical specimen, we employed recombinantly expressed TALEs fused to a fluorescent protein. For maximal signal/noise and minimal excess binding energy potentially compromising single‐nucleotide selectivity, we optimized the number of repeats per TALE. We evaluated TALEs of varying length targeting the SATIII consensus sequence “TGGAACGGAACGGAATGGAAT GGAATGGAA” by microscopy of stained HeLa cells and electromobility‐shift assays (Figure 2 a,b and Supporting Information S1‐2), and proceeded with a 17 repeat TALE, termed TALE_2 (for two CpGs in the target, Figure 1 b). We initially expressed two TALE_2 versions, bearing at CpG repeat positions 5 and 10 either an HD RVD selectively binding C (and being blocked by 5mC), or the RVD G* binding any nucleobase (including 5mC,^20^ Figure 1 b). In vitro footprinting assays confirmed 5mC sensitivity of the HD TALE with nucleotide/strand resolution, and universal binding of the G* TALE (Figure 2 c and the Supporting Information).

**Figure 2:**
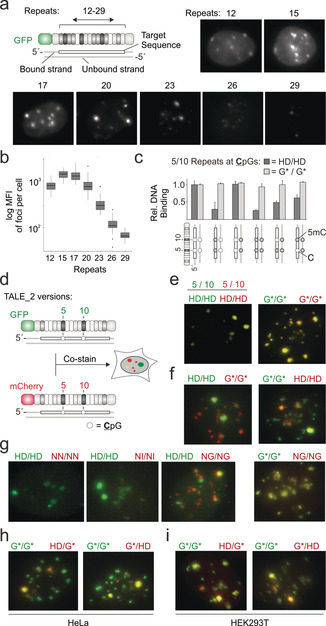
TALEs enable cellular DNA staining with single nucleotide selectivity. a) Optimization of TALE length by SATIII staining in HeLa cells. b) Mean fluorescence intensities (MFI) of foci per cell (N=512 cells) from stains of Figure 2 a. c) Relative binding of TALE_2 versions bearing HD or G* repeats opposite two **C**pG to DNA with different C/5mC patterns at the CpGs of the TALE‐target in the bound strand (white box) and opposite strand (from DNaseI footprinting, see Supporting Information). d) TALE_2 versions used for imaging in Figure 2 e–i. e) HeLa co‐stains using TALE_2s with indicated repeats at positions 5 and 10. f–h) Co‐stains as in Figure 2 e with indicated repeats. i) Stains as in Figure 2 h with HEK293T cells.

To study the actual nucleobase composition of SATIII DNA at the two target CpGs in the target sequence population, we co‐stained HeLa cells with GFP and mCherry fusions of the two TALE_2 versions (Figure 2 d). Co‐stains with either both HD TALEs or both G* TALEs showed full co‐localization, demonstrating that the fluorophores did not influence TALE selectivity (Figure 2 e and Figures S3 a and S4). Interestingly, despite similar affinities of HD and G* repeats14e, 20 (Figure S1 c), we observed more foci for the G* TALE, revealing a SATIII population not containing cytosine at the target CpG. Indeed, mixed co‐stains with HD and G* TALEs enabled visible separation of these two populations (Figure 2 f and Figure S3 a and S4). To reveal the actual nucleobases at these positions, we performed co‐stains with the HD TALE_2, and versions bearing the natural repeats NN, NI or NG (binding G, A and T/5mC, respectively; Figure 1 b and Figure S3 b and S5). Whereas the NN and NI TALEs did not afford foci, the NG TALE afforded many foci that fully co‐localized with the G* TALE (Figure 2 g). This indicates that the SATIII loci contain either C or 5mC/T at the target CpGs. These can be visibly separated, providing a suitable target for analysis of differential C/5mC levels. The results also reveal a high selectivity of natural TALE repeats in our staining procedure.

As prerequisite for 5mC analysis with single‐nucleotide resolution, we conducted co‐stains with G* TALE_2 and versions with one G* repeat replaced by HD, since this allows us to ignore one CpG in the TALE target sequence (by G*) and selectively interrogate the other (by HD).^21^ These afforded the same patterns as co‐stains with G* only and HD only TALEs (Figure 2 h, compare with Figure 2 f right), showing selectivity for single C versus 5mC/T differences in HeLa cells (and HEK293T cells, Figure 2 i and Figure S6). The absence of HD TALE fluorescence at many G* TALE foci thereby demonstrates the high selectivity of HD TALEs for single C‐positions, whereas the mixed fluorescence at other foci indicates that the C versus 5mC/T heterogeneity of CpG nucleobase compositions can be studied (Figure 2 h,i).

Next, we aimed at studying methylation changes at single CpG. For in vivo methylation of TALE targets with minimal perturbation of the global 5mC landscape, we constructed “DNMT^act^” consisting of DNMT3a3L^22^ fused to a TALE targeting the SATIII sequence “TGATTCCATTCCATTCCATT” (TALE_0, for zero CpG in target, Figure 3 a). This sequence differs from the target sequences of TALE_2 and other TALEs used for later staining to avoid competition. Bisulfite PCR and pyrosequencing revealed at a critical SATIII CpG a circa 6‐fold increased methylation for HEK293T cells expressing DNMT^act^ compared to a catalytically inactive E756A mutant^23^ (“DNMT^inact^”, Figure 3 b). Importantly, DNMT^act^ and DNMT^inact^ exhibited identical transfection and expression levels (Figure 3 c,d). Together with the identical DNA affinities of DNMT3a wt and E756A,^23^ this rules out the possibility of differential competition with TALEs in subsequent stains. Finally, TALE_0 of DNMT^act^/DNMT^inact^ extensively co‐localized with TALEs used for later analyses, providing many loci with expected differential methylation in DNMT^act^ versus DNMT^inact^ cells that can be studied with these TALEs (Figure 3 e).

**Figure 3:**
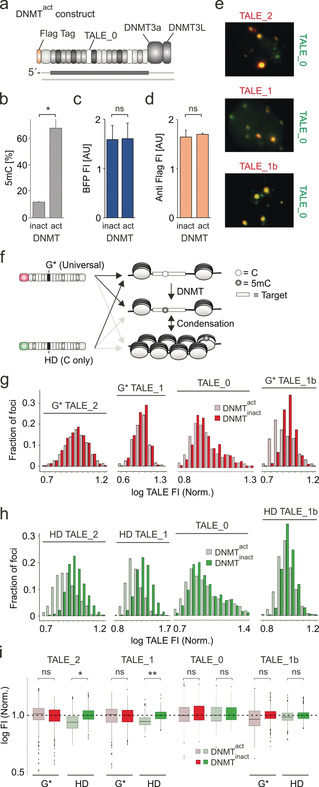
TALEs enable 5mC analysis at user‐defined CpG in HEK293T cells by imaging. a) Features of DNMT^act^. b) Pyrosequencing analysis of SATIII target CpG methylation for DNMT^act^/DNMT^inact^ cells (paired *t*‐test; *N*=3, *=*p*<0.05). c) Flow cytometry analysis of BFP transfection control FI (fluorescence intensity) from DNMT vectors and d) of DNMT expression itself through flag immunostain. *N*=4 and 4, ns=not significant, AU=arbitrary units. e) Co‐stains with mCherry‐TALEs and GFP‐TALE_0. f) Expected binding of G* and HD TALEs to different target states. g) Histogram of G* TALE FI of foci from DNMT^act^/DNMT^inact^ cells co‐stained with HD and G* TALEs. For each TALE, log FI of each foci are normalized to the mean of log FI of all foci of DNMT^inact^ cells. h) Same for HD TALEs. i) Box plots of data from (g,h). Paired *t*‐test with *N*=5, 6, 3, and 6; and >1400 foci; *=*p*<0.05; **=*p*<0.01.

Arguably, the binding of TALEs designed for differentially methylated CpGs could be affected by differences in target DNA accessibility rather than in 5mC itself. We fancied, whether we could account for this by designing TALE pairs consisting of a GFP‐TALE with HD repeats, and a control mCherry‐TALE with G* repeats opposite the targeted C‐positions. In co‐stains, a negative response only of the HD TALE would reveal increased 5mC, whereas negative response of both TALEs would reveal decreased overall target accessibility (Figure 3 f).

We co‐stained DNMT^act^ or DNMT^inact^‐transfected HEK293T cells with G* and HD TALE_2 versions, and recorded signals for foci showing both mCherry and GFP fluorescence. For comparability, we normalized for each TALE the signals to the mean of the DNMT^inact^ signals. For the G* TALE, the foci showed highly similar fluorescence in both cell types, indicating similar target accessibility (Figure 3 g,i). In contrast, the HD TALE fluorescence was markedly reduced in DNMT^act^ cells, indicating a selective response to increased 5mC (Figure 3 h,i). The same was true for a TALE targeting the alternative sequence “TGGAATCAACCCGAGTA”, confirming this effect for a different target containing only one CpG (TALE_1, Figure 3 g–i). Strikingly, TALE_0 that targets a CpG‐free, non‐methylatable sequence showed no difference for cells expressing DNMT^act^/DNMT^inact^ constructs (Figure 3 g–i and the Supporting Information; note that TALE_0 exists only as HD version with either fluorophore, since not targeting a CpG). Finally, we observed for a TALE targeting another single‐CpG sequence (TGGAATCAACACGAGTGG; TALE_1b) a trend of reduced fluorescence in DNMT^act^ cells for both G* and HD versions. This suggests that methylation of this sequence was indeed associated with reduced target accessibility.

We next aimed to study the role of 5mC in the regulation of nSB formation. This stress response mechanism is initiated by recruitment of heat‐shock factor 1 (HSF1) to SATIII DNA, triggering transcription of the long non‐coding RNA SATIII. This induces nSB formation and sequestration of for example, splice factors as pathway for global translational down‐regulation (Figure 4 a).^17^ HSF1 recognizes an nGAAn consensus sequence often preceded by CpG, raising the possibility of 5mC control. Indeed, SATIII is hypomethylated and over‐transcribed in several cancers and can be induced by 5‐azacytidine in HeLa cells.^18^ However, functional loss of DNMT in other cell types does not induce SATIII, suggesting multilayered regulation.^24^ To study the interplay of 5mC and HSF1 at individual SATIII loci of single cells, we combined TALE‐imaging with HSF1 immunostainings of U2OS bone cancer cells that exhibit strong HSF1 recruitment upon heat shock (Figure 4 b). We grew DNMT^act^ and DNMT^inact^ cells under heat‐shock conditions and co‐stained them with HD and G* TALE_2, and with an antibody against endogenous HSF1. We observed reduced binding of the HD but not the G* TALE, indicating differential target methylation with unaltered overall accessibility (Figure 4 c and Figures S11 and S12). Interestingly, we observed a weakly increased HSF1 recruitment for DNMT^act^ foci (Figure 4 c). Histogram analysis revealed that this was due to a population of cells with high HSF1 (Figure 4 d). To study the influence of 5mC on HSF1 recruitment, we recorded all three fluorescence signals for each focus. We then plotted the HSF1 FI versus the ratio of G* to HD TALE FIs (as a measure of methylation) as means per cell. Indeed, we found a population of cells with particularly high HSF1 recruitment in DNMT^act^ cells (Figure 4 d) that also showed higher G* to HD TALE FI ratios (Figure 4 e). This argues for a positive role of 5mC in heat shock‐dependent HSF1 recruitment in U2OS cells that can be studied on the level of individual foci and cells by our TALE approach.

**Figure 4:**
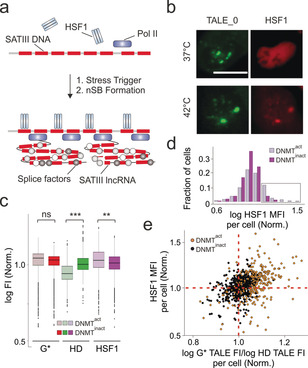
TALEs reveal a role of 5mC in the regulation of heat‐shock‐induced recruitment of HSF1. a) Scheme of nSB formation. b) Imaging of U2OS cells expressing mClover3‐TALE_0 and mCherry‐HSF1 with or without heat‐shock. c) Foci FI from DNMT^act^/DNMT^inact^ cells co‐stained with HD and G* TALE_2, and anti‐HSF1 antibody. Data normalized as in Figure 3 g–i. Paired *t*‐test with *N*=7 experiments totaling 990 cells; **=*p*<0.01; ***=*p*<0.001; ns=not significant. Further foci/cell data/statistics in the Supporting Information. d) Histogram of HSF1 FI of foci from (c) analysed per cell. High HSF1 cells in box. e) Scatter plot of HSF1 versus G* TALE/HD TALE MFI of foci for each cell from (d).

In summary, we report TALEs as programmable receptors for the imaging‐based analysis of single 5mC positions in user‐defined DNA sequences of single cells. We employ pairs of one TALE with C‐selective repeat and one with universal repeat opposite the target C‐position in co‐stains to analyze 5mC independently of differences in overall target accessibility. Combination with immunostaining enables correlations between 5mC and HSF1, revealing a positive role of 5mC in heat‐shock‐induced HSF1 recruitment. For studying dynamic processes, we are currently extending our approach to live cell imaging by protein transfection of TALE pairs. Taken together, our study demonstrates that programmable receptors with selectivity beyond A, G, T and C open new avenues to study roles of epigenetic DNA modifications in shaping chromatin functions in situ, with nucleotide, locus, and cell resolution.

## Conflict of interest

The authors declare no conflict of interest.

## Supporting information

As a service to our authors and readers, this journal provides supporting information supplied by the authors. Such materials are peer reviewed and may be re‐organized for online delivery, but are not copy‐edited or typeset. Technical support issues arising from supporting information (other than missing files) should be addressed to the authors.

Supplementary
