# Supplementary material for: Designer Receptors for Nucleotide‐Resolution Analysis of Genomic 5‐Methylcytosine by Cellular Imaging
Source: Angew Chem Int Ed Engl. 2020 Apr 7;59(23):8927–31. doi: 10.1002/anie.202001935 (PMC7318601; doi:10.1002/anie.202001935)
Supplement: Supplementary file 1 — Supplementary [file ANIE-59-8927-s001.pdf]

Angewandte Chemie
Eine Zeitschrift der Gesellschaft Deutscher Chemiker

Supporting Information

Designer Receptors for Nucleotide-Resolution Analysis of Genomic 5-Methylcytosine by Cellular Imaging

Álvaro Muñoz-López, Benjamin Buchmuller, Jan Wolffgramm, Anne Jung, Michelle Hussong, Julian Kanne, Michal R. Schweiger, and Daniel Summerer**

anie_202001935_sm_miscellaneous_information.pdf

Table of Contents

Material and Methods

- Plasmid Cloning S3
- Cell Culture and Transfection S4
- Flow Cytometry and Cell Sorting S4
- TALE expression and purification S4
- TALE staining S5
- Co-staining with TALEs and HSF1 antibody S6
- FLAG-tag immunostaining S6
- Bisulfite conversion S6
- Pyromark PCR S7
- Pyrosequencing S7
- Electromobility Shift Assay S7
- Microscopy S7
- Image Processing and Analysis S8
- Data Analysis and Statistics S8
- Oligo Table S10
- TALEs Assembly Table S11

Supplementary Figures

- Figure S1 .... S12
- Figure S2 .... S13
- Figure S3 .... S15
- Figure S4 .... S17
- Figure S5 .... S18
- Figure S6 .... S19
- Figure S7 .... S21
- Figure S8 .... S23
- Figure S9 .... S24
- Figure S10 .... S25
- Figure S11 .... S27
- Figure S12 .... S28

Supplementary References S30

Appendix 1 – Vector Maps S31

MATERIAL AND METHODS

Plasmid cloning

Plasmid pÁlM1577 for expression of TALEs fused to N-terminal mCherry (for vector maps, see Appendix 1) was generated by amplifying mCherry from pAnW1272 using primers o2994 and o2995 (see oligo table for primer sequences). PCR fragment and vector pAnI521[1] were digested with NdeI and BsrGI enzymes and then ligated with T4 ligase (New England Biolabs, M0202T) using 3:1 insert:vector molar ratio.

pJaW876 was cloned by Gibson assembly[2]. For this, vector pcDNA3.1-GoldenGate-VP64 (Addgene, #47389) was linearized by PCR using primers o2079 and o2080 and the insert DNMT3a3L CD was amplified by PCR using pET28-Dnmt3a3L-sc27 (Addgene, #71827) and o2071 and o2072 primers.

Vector pÁlM1285 was created by amplifying CMV-EBFP2 from plasmid EBFP2-N1 (Addgene #54595) using primers o2733 and o2734. Vector pJaW876 was digested using MfeI and previously amplified CMV-EBFP2 fragment was inserted by Gibson Assembly resulting in pÁlM1285. Mutation E756A for catalytically inactive DNMT3a3L was introduced by site-directed mutagenesis using primers o2038 and o2039 to generate pÁlM1560 vector.

Library of G* RVD modules was prepared as follows: pG*1 module was generated by QuikChange site-directed mutagenesis (Agilent) using o2166 and o2167 primers with pHD1 vector. Modules pG*2 to pG*9 were derived from pHD2 to pHD9[3] module vectors by site-directed mutagenesis using o2168 and o2169 primers. Vector pG*10 was created by cassette mutagenesis by digesting pHD10 with NcoI-HF and BsoBI restriction enzymes and ligating the double strand fragment generated by annealing o2142 and o2143 oligos.

TALEs were assembled by Golden Gate Assembly as previously described[3] (see TALEs assembly table for detailed RVD composition) using pAnI521 or pÁlM1577 as entry plasmids in Golden Gate 2 reactions, resulting in plasmids coding for the respective TALE proteins in frame with a C-terminal His6x tag and a N-terminal eGFP or mCherry, respectively. For mammalian live-cell imaging, TALEs were assembled in vector pTALYM3 (Addgene, #47874). For site-specific DNA methylation, TALEs were assembled in p1285 or p1560 for TALE-DNMT^{active} or TALE-DNMT^{inact} fusions, respectively.

HSF1 gene was amplified from HEK293T cDNA with primers HSF1-Bgl1-fw and HSF1-Sal1-rv. For cloning into pmCherry–C3[4], vector and insert were digested with Sal1 and Bgl1, and re-ligated in presence of the inserts with T4 Ligase at 16°C, overnight in order to obtain pHSF1-mCherry.

Cell culture and transfection

HEK 293T, HeLa and U2OS (Sigma Aldrich, 92022711-1VL) cells were cultured at 37ºC and 5% of CO_2 in DMEM medium (PanBiotech, P04-03609) supplemented with 10% FBS (PanBiotech, P30-3302), 1% of L-Glutamine 200mM (PanBiotech, P04-80100) and 1% of Pen/Strep (PanBiotech, P06-07050).

Cells were seeded on 10 cm diameter dishes (Sarstedt, 83.3902.300) and transfected with 10 μg of $DNMT^{act}$ or $DNMT^{inact}$ (TALE_0 and TALE_1C assembled in p1285 and p1560) plasmid using FuGene 6 Transfection Reagent (Promega, E2691) following the manufacturer's protocol. The reagent:DNA ratio used was 3:1 in every case. For HSF1 and SatIII-TALEs co-staining in live cells, 300.000 U2OS cells were seeded on a μ-Dish 35 mm (ibidi, 81156) and transfected with 1 μg of TALE_0 assembled in pTALYM3 vector and 500 ng of pHSF1-mCherry as mentioned above.

Flow cytometry and cell sorting

Cells were washed with DPBS, trypsinized with Trypsin 0.05% / EDTA 0.02% (PanBiotech, P10-038100) for 5 minutes at 37ºC and blocked with Full DMEM medium. Samples were pelleted by centrifugation at 300 g for 10 minutes and then resuspended in DPBS for flow cytometry.

Cells were sorted with a Sony Cell Sorter model LE-SH800SFP in targeted mode using the 405nm laser (filter FL1 450/50, Optical Filter Pattern 2) in order to detect EBFP2 from DNMT-TALE plasmids. Gates were set to assure similar expression levels of fluorescent protein in both: samples transfected with $DNMT^{act}$ or $DNMT^{inact}$ plasmids (TALE_0 and TALE_1C assembled in p1285 and p1560). EBFP2+ cells were collected in tubes with Full DMEM + 2% of Pen/Strep (PanBiotech, P06-07050), counted and seeded in μ-Plate 96 Well Black ibiTreat, tissue culture treated plates (ibidi, 89626) previously coated with 0.02% of poly-L-Lysine (Sigma Aldrich, P1274-100MG). Multicolor flow cytometry was compensated using single stained samples for each fluorophore.

TALE expression and purification

TALEs were expressed and purified as previously described[5]. Briefly, TALE plasmids were transformed in electrocompetent BL21 DE3 Gold *E.coli* cells and grown overnight at 37ºC on carbenicillin (Carb, 100 mg/ml) LB agar plates. Single colonies were picked and grown in 5 mL LB supplemented with 100 mg/ml of carbenicillin for 3 hours at 37ºC and 220 r.p.m. This starter culture was transfer to a flask containing 250 mL of LB + Carb and grown under the same conditions until the OD^{600} reached 0.8 arbitrary units (au), followed by 0.5 mM IPTG induction and incubation overnight at 22ºC and 220 r.p.m. Cultures were centrifuged at 3000 g and 4ºC for 20 minutes. Supernatant was

discarded and pellets were kept at -20ºC during 2 hours. Pellet was resuspended in 10 mL of Deep Lysis Buffer (10 mM Tris-HCl, 300 mM NaCl, 2,5 mM MgCl2, 5 % DMSO, 0.2 % sodium lauroyl sarcosinate (AppliChem), 0.1 % Triton X-100, pH = 9) containing 1 mM PMSF, 1 mM of DTT and 50 μg/mL lysozyme (Sigma Aldrich). The suspension was lysed by repeated sonication on ice (2 runs of 3 minutes; 20 % amplitude; 4s on, 2s off), letting samples cool on ice for 1 minute between runs. Samples were centrifuged at 14000 g and 4ºC for 20 minutes to remove cell debris. Supernatant was then incubated with 1 ml HisPur™ Ni-NTA Resin (ThermoFisher Scientific, 88221) overnight spinning at 4ºC. Beads were then washed twice with PBS, three times with Lysis buffer + 20 mM Imidazole + 1mM DTT and twice with Lysis buffer + 50 mM Imidazole + 1mM DTT. Elution of the TALEs was performed by incubating the beads with 1mL of Lysis buffer + 500 mM Imidazole + 1mM DTT overnight. Samples were centrifuged at 12000 g for 5 minutes. Supernatant was taken and load into a dialysis cassette (Thermo Scientific, 66381). Samples were dialyzed in TALE Storage Buffer (200 mM NaCl, 20 mM Tris, 10% glycerol, pH = 7.5) + 1 mM DTT stirring at 4ºC and exchanging the buffer every 2 hours for 5 times. Last dialysis step was performed overnight. Samples were centrifuge at 12.000 g and 4ºC for 5 minutes and supernatant was aliquot, snap-froze with liquid nitrogen and stored at -80ºC. Protein concentration was measured by BCA using Microplate BCA Protein Assay Kit – Reducing Agent Compatible (ThermoFisher Scientific, #23252) following manufacturer's instructions.

TALE staining

20.000 HEK 293T or HeLa cells or 17.000 U2OS cells per well were seeded on a μ-Plate 96 Well Black ibiTreat tissue culture treated plates (ibidi, 89626). Cell were washed once with DPBS (PanBiotech, P04-361000) and then fixed with ice-cold methanol at -20ºC for 10 minutes. Cells were washed again with DPBS for 5 minutes at 300 rpm and then treated with HCl 2N for 5 minutes at room temperature. Cells were washed twice with DPBS and blocked with DPBS-T + 1% BSA overnight. Samples were then incubated with 200 μl 0.8 nM purified TALEs in TALE Binding Buffer (20 mM Tris-HCl pH= 8, 50 mM NaCl, 5 mM $MgCl_2$, 50 ng/μl Salmon Sperm DNA, 0.1 mg/ml BSA and 10% glycerol) for TALE_1, TALE_1b and TALE_0 and DPBS + 50mM of NaCl for TALE_2 at room temperature for 30 minutes. Each well was washed four times with the respective buffer for 5 minutes at room temperature and shaking at 300 rpm. Plates were kept in DPBS + 5% FBS overnight at 4ºC. Nucleus staining was performed by incubating the samples with 1 uL per well of Vectashield with DAPI in 200 μl of DPBS (Vector Laboratories, H-1200) for 10 minutes at room temperature. Finally, cells were washed twice with the respective buffer for 5 minutes at room temperature and kept in DPBS for microscopy.

Co-staining with TALEs and HSF1 antibody

Cells were incubated under heat-shock conditions for 2 hours at 43ºC and immediately after, they were washed with DPBS and then fixed with formaldehyde 1% for 5 minutes at room temperature, followed by 10 minutes at 4ºC. After washing three times with DPBS-T (0.05% Tween-20), samples were incubated with ice cold methanol for 10 minutes at -20ºC and then washed again. Cells were blocked with blocking buffer (DBPS with 0.05% of Tween-20 and 1% of BSA) overnight at 4ºC and then treated with HCl 2N for 5 minutes at room temperature. Samples were thoroughly washed with DPBS-T three times and then incubated with 1:500 dilution of anti-HSF1 antibody (Cell Signaling Technology, 4356S) in blocking buffer for 1 hour at room temperature, followed by three washes and incubation with 1:250 dilution of Alexa Fluor 750 secondary antibody (Invitrogen, A21039) under the same conditions. After washing, cells were incubated with TALEs as described above. Each well was washed four times with the respective buffer for 5 minutes at room temperature and shaking at 300 rpm. Finally, nucleus staining was performed as described above.

FLAG-tag immunostaining

HEK 293T cells were trypsinized for 5 minutes at 37ºC, centrifuged down and fixed using the FIX & PERM Cell Permeabilization Kit (ThermoFisher Scientific, GAS003) following manufacturer's instruction. Then samples were first blocked with 1% of BSA in DPBS-T for 1 hour at room temperature and then incubated with primary anti-FLAG (Sigma-Aldrich, F1804-200UG) in blocking buffer for 1 hour at room temperature. After washing three times, staining with secondary Alexa Fluor 488 antibody (Invitrogen, A11001) was performed under the same conditions. Cells were washed and resuspended in FACS buffer (DPBS + 2% FBS + 2 mM EDTA) for flow cytometry.

Bisulfite conversion

For the bisulfite conversions the EpiTect® Bisulfite Kit (QIAGEN, 59104) was performed according to the protocol of the manufacturer. In brief, for each reaction 200 – 250 ng DNA was used. After the bisulfite conversion the solution was loaded onto EpiTect spin columns and centrifuged at maximum speed (13,400 x g). After discarding the flow through and washing, 500 µL of desulfonation buffer was applied to each column. After 15 minutes of incubation at room temperature, the columns were centrifuged again for one minute at max. speed. After two further washing steps and one dry centrifugation the samples were eluted in 20 µL of elution buffer.

Pyromark PCR

Pyromark PCR was done with the Pyromark PCR kit (QIAGEN, 978703). The reaction mixture for the PCR was prepared according to the manufacturer's protocol. A final primer concentration of 0.2 μM of oligos SatIII-Pyro-fw and SatIII-Pyro-rv was applied and 10 ng of template DNA were added.

Pyrosequencing

The Pyromark PCR product was bound to sepharose beads on a 96-well plate utilizing the biotinylation of either forward or reverse primers. The bound PCR products were shaken on a plate shaker for five minutes at room temperature. In the meantime, 10 µM of sequencing primers were diluted in annealing buffer and added onto a separate PSQ 96-well plate. A vacuum filter station was then used for the washing of the PCR product bound to the sepharose beads. The filters were first washed with water before being immersed into the DNA solution on the first 96-well plate. The filters were then immersed into 70% ethanol, 0.2 M NaOH and washing buffer containing 10 mM tris-acetate (pH 7.6). Afterwards, the vacuum was turned off and the filters were placed into the PSQ 96-well plate containing the diluted sequencing primers, which led to the detachment of the sepharose beads from the filters. After letting the filters stand in the solution for several minutes, the plate, now containing both, the primers as well as the DNA templates, was placed on a heating block for 2 minutes at 85°C. After the heating, the plate was kept at room temperature. The pyrosequencing reaction was performed in a PSQ HS 96ATwo Pyrosequencer and analysed using thw PSQ HS 96A software.

Electromobility shift assay (EMSA)

Electromobility Shift Assays were performed as previously reported[6] using Cy5-labelled oligo o3546 and o3552. Briefly, pairs of oligos were hybridized in Annealing Buffer (20 mM Tris, 50 mM NaCl, 5 mM MgCl2 and 5% v/v glycerol, pH = 8) by incubating for 5 minutes at 95ºC, followed by 30 minutes at room temperature. Then, 7.5 pmol of annealed oligos were incubated with 1.5 pmol of TALEs in TALE Binding Buffer (see above) for 1 hour at room temperature and 30 minutes at 4ºC. In case of competitive EMSA, equimolar amounts (1.5 pmol) of both HD-EGFP and G*-mCherry TALEs were incubated with unlabeled annealed oligos. The native polyacrylamide gel (0.5 x TAE buffer, 8 % Rotiphorese gel 40 (Carl-Roth), 0.1 % APS and 0.01 % TEMED) was pre-run at 4 °C for 30 minutes in a Mini Protean vertical electrophoresis cell (Bio-Rad) at 70 V. The gel was loaded with 7,5 µl sample and then run for 90 minutes at 4 °C and 70 V. GFP fluorescence of the gels was recorded with a Typhoon FLA-9500 laser scanner (GE Healthcare) and analyzed using the software ImageQuant TL 8.1 (GE Healthcare).

Microscopy

Images were taken as z-stacks of 6 μm (step size = 0.3μm) of three channels using a 60X oil objective with an Olympus IX81 microscope coupled with Hamamatsu model C10600-10B-H camera. Immersion oil was ibidi immersion oil (ibidi, 50101) compatible with μ-Plate 96 Well Black ibiTreat, tissue culture treated plates. DAPI was acquired using the DAPI excitation filter (387/11), EGFP with excitation filter GFPFret (470/22) and mCherry with Cy3(560/25). All the channels were used with triple band dichroic DaFICy3 cube and the DaFICy3 quad band emission filter. When using Alexa Fluor 750, a fourth channel was added and images were acquired using Cy7 HC Filterset cube (F36-577) and Cy5-Cy7 emission filter (ET700/75). Exposure times were 30, 100 and 50 ms for DAPI, EGFP and mCherry respectively. For HSF1 and TALEs co-staining 30, 200, 100, 100 ms exposure times were used for DAPI, EGFP, mCherry and Alexa Fluor 750, respectively.

Image processing and analysis

The intensity and subcellular localization of foci was analyzed from maximal intensity Z-projections of image stacks (1344 x 1024 pixels, 12 bits) using the Fiji distribution of ImageJ[7,8] as follows: The mean background intensity of an out-of-interest region was subtracted from each channel of the stack, and the nuclear regions selected from the DAPI channel (10 μm^2 minimum area, circularity between 0.5–1.0). We then used the "GaussFit OnSpot" plugin[9,10] to analyze the intensity and size of the spots recorded in the GFP/FITC using elliptical shape and Levenberg Marquard fit mode with a 10 pixel rectangle half size. In order to select foci-like objects from this set, spots outside the nuclear regions or with a prominence smaller than 30 (signal-to-noise ratio) or spots larger than 25 pixel were excluded. Mask (ROIs) generated from GFP/FITC channel were directly applied to mCherry and Cy7 channels to measure the mean fluorescence intensity. The images were processed in batch using an ImageJ macro script. For each nucleus, the number, size and intensity of the associated foci was recorded.

Data analysis and statistics

The data was analyzed and plotted using R[11,12]. DAPI areas larger than 25000 square pixels were filtered out to exclude unsplit nuclei. For each TALE, mean fluorescence intensities of each foci were log transformed and then normalized by the average fluorescence intensity of all foci of the $DNMT^{inact}$ sample of each independent experiment. Single cell data was performed by calculating the average mean fluorescence intensity of all foci within a single nucleus. Single cell data was log transformed and normalized as explained above. Graphs were plotted using ggplot2 library[13] and statistical analyses were performed using GraphPad to carry out paired t-test considering number of independent experiments as sample size ($N \geq 3$ independent experiments in every case). Statistical t-test analyses

by cell and by foci were performed with ggpubr[14] (unpaired). Significance of t-test studies and sample sizes for each experiment are shown in the tables below:

Sample Size			
	N (# of experiments)	# of cells	# of foci
TALE_2	5	751	2560
TALE_1	6	842	1484
TALE_1b	6	869	2764
TALE_0	3	248	1444
HSF1 experiment	7	990	2969

	**Significance for paired t-test**								
	**Biological Replicates**			**Cells**			**Foci**		
	**HD**	**G***	**HSF1**	**HD**	**G***	**HSF1**	**HD**	**G***	**HSF1**
**TALE_2**	*	ns	-	****	ns	-	****	ns	-
**TALE_1**	**	ns	-	****	ns	-	****	ns	-
**TALE_1b**	ns	ns	-	****	****	-	****	****	-
**TALE_0**	ns	ns	-	ns	ns	-	ns	ns	-
**HSF1 experiment**	***	ns	**	****	ns	***	****	ns	****

Oligos table

Oligo name	Sequence (5' → 3')
o2994	TATACATATGGTGAGCAAGGGCGAGGAG
o2995	AGCGTAGTCCGGAACGTCG
o2079	ATTAACTACCCGTACGACGTTC
o2080	GGCGCGCCCAACTTTGCGTTT
o2071	AAACGCAAAGTTGGGCGCGCCAACCATGACCAGGAATTTGACC
o2072	GAACGTCGTACGGGTAGTTAATAAGAGGAAGTGAGTTTTGAG
o2733	GAGCAAAATTTAAGCTACAACAAGGCAAGGCTTGACCGACGCGTTACATAACTTACGGTAAATGG
o2734	AGCAGCGCAAAACGCCTAACCCTAAGCAGATTCTTCATGCGCTTACTTGTACAGCTCGTCC
o2038	CCCTTCTTCTGGCTCTTTGCCAATGTGGTGGCCATGGGCG
o2039	CCATGGCCACCACATTGGCAAAGAGCCAGAAGAAGGGGCG
o2166	GGTCTCGCTATCGCCAGCGGCGGCGGCAAG
o2167	GCCGCCGCCGCTGGCGATAGCGAGACCTC
o2168	GCTATCGCCAGCGGCGGCGGCAAGCAAGCG
o2169	TTGCTTGCCGCCGCCGCTGGCGATAGCCAC
o2142	CATGGCCTGACTCCGGACCAAGTGGTGGCTATCGCCAGCGGAGGCGGGAGACCC
o2143	TCGAGGGTCTCCCGCCTCCGCTGGCGATAGCCACCACTTGGTCCGGAGTCAGGC
HSF1-Bgl1-Fw	TTTAAGTAGATCTGATCTGCCCGTGGGCCCCGG
HSF1-Bgl1-Rv	TTTAAGTGTCGACCTAGGAGACAGTGGGGTCC
SatIII-Pyro-Fw	GGAATGGATTCAACTTGAATG
SatIII-Pyro-Rv	TTCCATTCCATTCCTATACT
o3552	CCAATGGAACGGAACGGAATGGAATG
o3546	NCATTCCATTCCGTTCCGTTCCATTGG N=Cy5

TALE assembly

TALE_2 Length Optimization

	1	2	3	4	5	6	7	8	9	10	11	12	13	14	15	16	17	18	19	20	21	22	23	24	25	26	27	28	29
29	NN	NN	NI	NI	HD	NN	NN	NI	NI	HD	NN	NN	NI	NI	NG	NN	NN	NI	NI	NG	NN	NN	NI	NI	NG	NN	NN	NI	NI
25	NN	NN	NI	NI	HD	NN	NN	NI	NI	HD	NN	NN	NI	NI	NG	NN	NN	NI	NI	NG	NN	NN	NI	NI	NG				
23	NN	NN	NI	NI	HD	NN	NN	NI	NI	HD	NN	NN	NI	NI	NG	NN	NN	NI	NI	NG	NN	NN	NI						
20	NN	NN	NI	NI	HD	NN	NN	NI	NI	HD	NN	NN	NI	NI	NG	NN	NN	NI	NI	NG									
17	NN	NN	NI	NI	HD	NN	NN	NI	NI	HD	NN	NN	NI	NI	NG	NN	NN												
15	NN	NN	NI	NI	HD	NN	NN	NI	NI	HD	NN	NN	NI	NI	NG														
12	NN	NN	NI	NI	HD	NN	NN	NI	NI	HD	NN	NN	NI																

TALE_2 Selectivity

	1	2	3	4	5	6	7	8	9	10	11	12	13	14	15	16	17
HD/HD	NN	NN	NI	NI	HD	NN	NN	NI	NI	HD	NN	NN	NI	NI	NG	NN	NN
G*/G*	NN	NN	NI	NI	G*	NN	NN	NI	NI	G*	NN	NN	NI	NI	NG	NN	NN
NN/NN	NN	NN	NI	NI	NN	NN	NN	NI	NI	NN	NN	NN	NI	NI	NG	NN	NN
NI/NI	NN	NN	NI	NI	NI	NN	NN	NI	NI	NI	NN	NN	NI	NI	NG	NN	NN
NG/NG	NN	NN	NI	NI	NG	NN	NN	NI	NI	NG	NN	NN	NI	NI	NG	NN	NN
HD/G*	NN	NN	NI	NI	HD	NN	NN	NI	NI	G*	NN	NN	NI	NI	NG	NN	NN
G*/HD	NN	NN	NI	NI	G*	NN	NN	NI	NI	HD	NN	NN	NI	NI	NG	NN	NN

TALE_1

	1	2	3	4	5	6	7	8	9	10	11	12	13	14	15	16
HD	NN	NN	NI	NI	NG	HD	NI	NI	HD	HD	HD	NN	NI	NN	NG	NI
G*	NN	NN	NI	NI	NG	HD	NI	NI	HD	HD	G*	NN	NI	NN	NG	NI

TALE_1b

	1	2	3	4	5	6	7	8	9	10	11	12	13	14	15	16	17
HD	NN	NN	NI	NI	NG	HD	NI	NI	HD	NI	HD	NN	NI	NN	NG	NN	NN
G*	NN	NN	NI	NI	NG	HD	NI	NI	HD	NI	G*	NN	NI	NN	NG	NN	NN

TALE_0

	1	2	3	4	5	6	7	8	9	10	11	12	13	14	15	16	17	18	19
HD	NN	NI	NG	NG	HD	HD	NI	NG	NG	HD	HD	NI	NG	NG	HD	HD	NI	NG	NG

TALE_1c

	1	2	3	4	5	6	7	8	9	10	11	12
HD	NN	NN	NI	NI	HD	NN	NN	NI	NI	NG	NN	NN

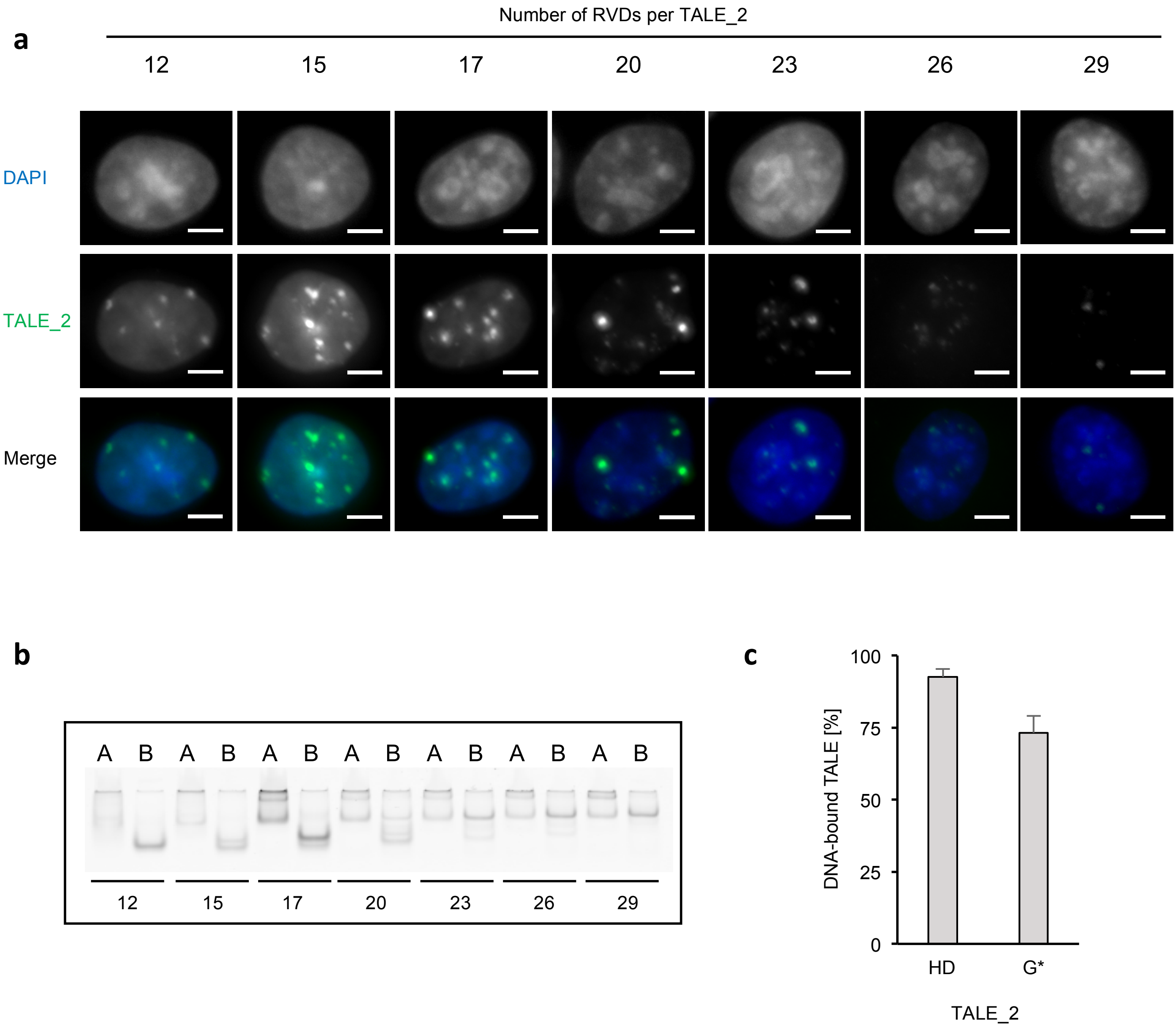

Figure S1. TALE Length Optimization. a) TALE stainings for length optimization using different truncations (29 – 12 RVDs, see TALEs Assembly table) of TALE_2 (2 CpGs) fused to EGFP in HeLa cells. Scale bar is 5μm. **b)** Electromobility Shift Assay of the versions of TALE_2. A indicates only TALE, B indicates that the TALE of the given length was used in combination with DNA. **c)** Competitive EMSA with equimolar concentrations of TALE_2-EGFP bearing HD and TALE_2-mCherry bearing G* at positions 5 and 10.

a

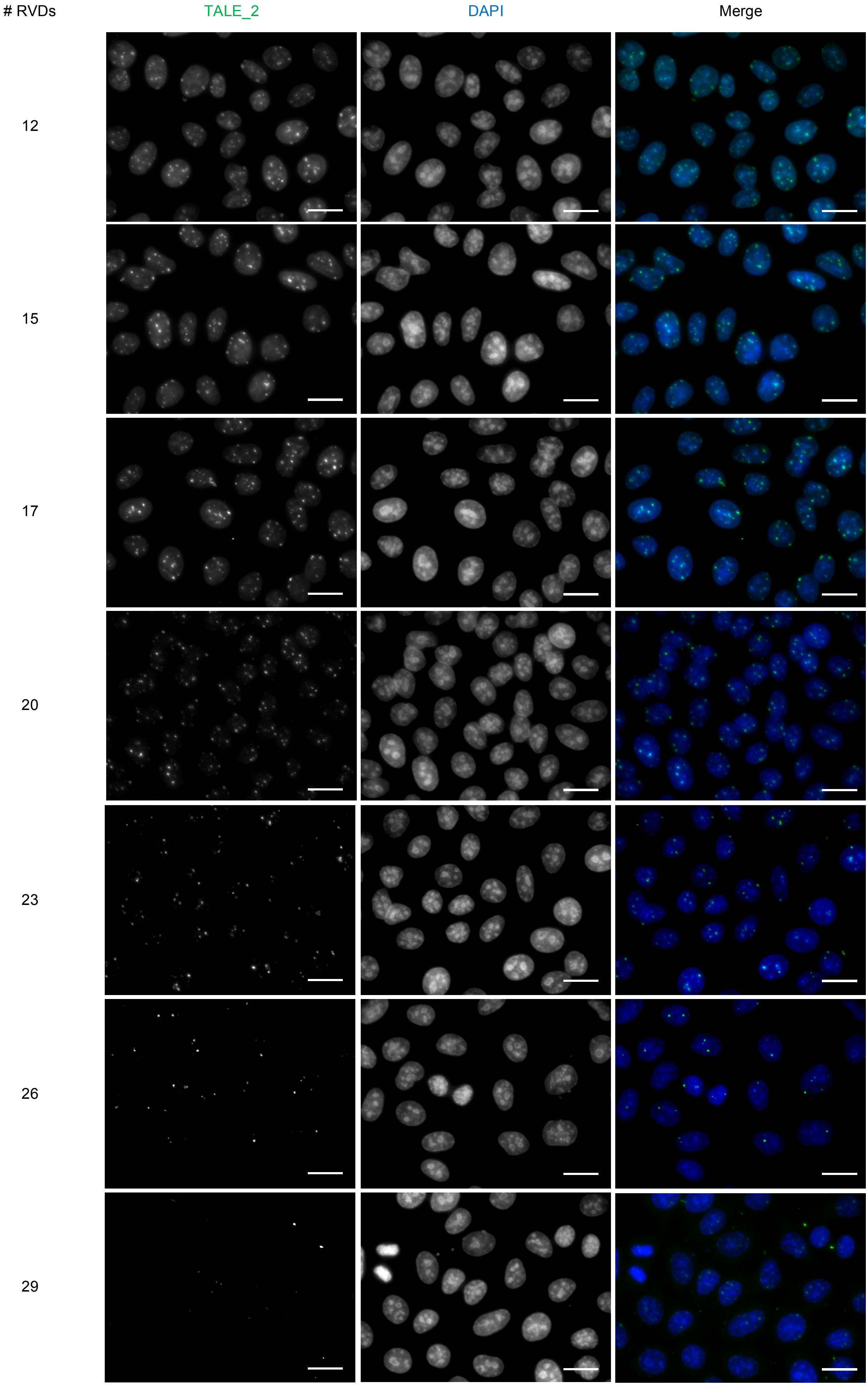

Figure S2. TALE Length optimization. a) TALE staining of different truncations of TALE_2 (12 – 29 RVDs) fused to EGFP in HeLa cells. Scale bar is 20 μm.

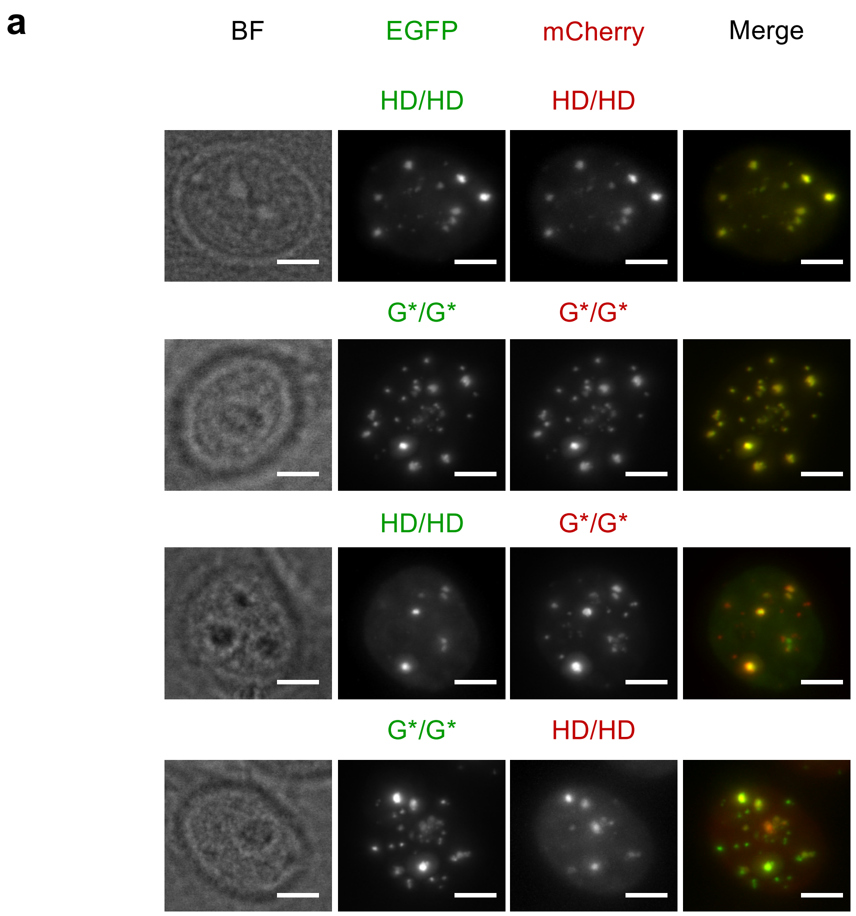
a
BF
EGFP
mCherry
Merge
HD/HD
HD/HD
G*/G*
G*/G*
HD/HD
G*/G*
G*/G*
HD/HD

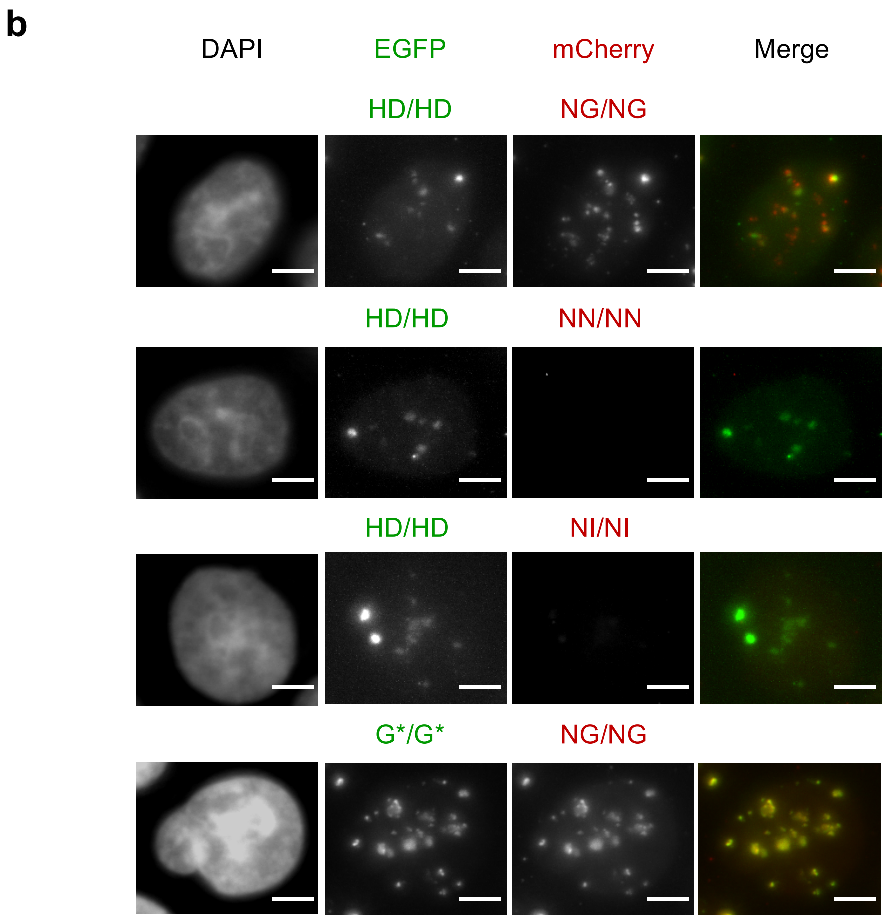
b
DAPI
EGFP
mCherry
Merge
HD/HD
NG/NG
HD/HD
NN/NN
HD/HD
NI/NI
G*/G*
NG/NG

Figure S3. TALEs selectivity. a) HeLa co-stains with TALE_2 bearing indicated repeats at positions 5 and 10. b) Co-stains as in Fig. S3 with indicated repeats. Scale bar is 5 μm.

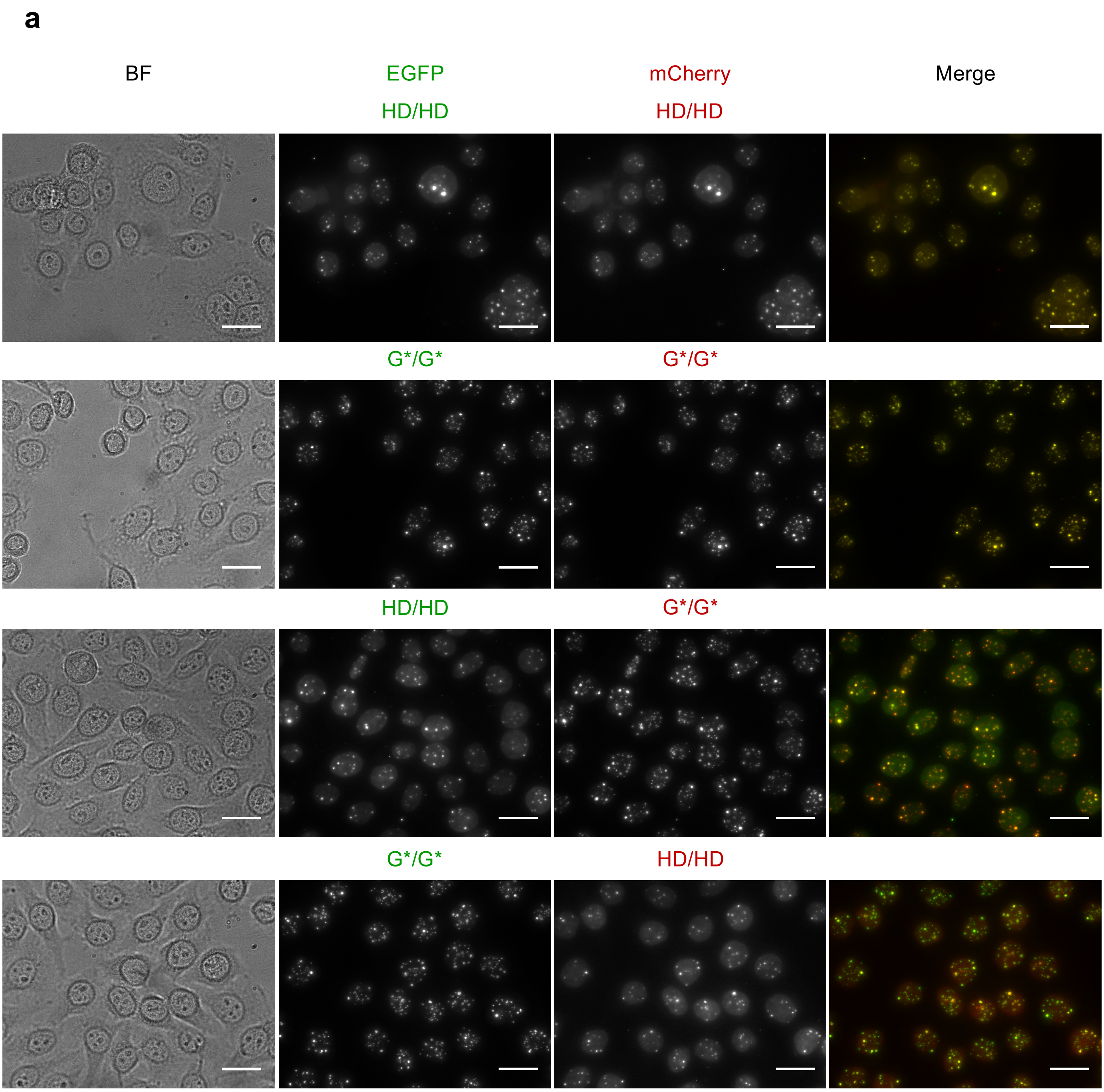

Figure S4. TALEs selectivity. a) HeLa co-stainings with TALE_2 bearing indicated repeats at positions 5 and 10. Scale bar is 20 μm.

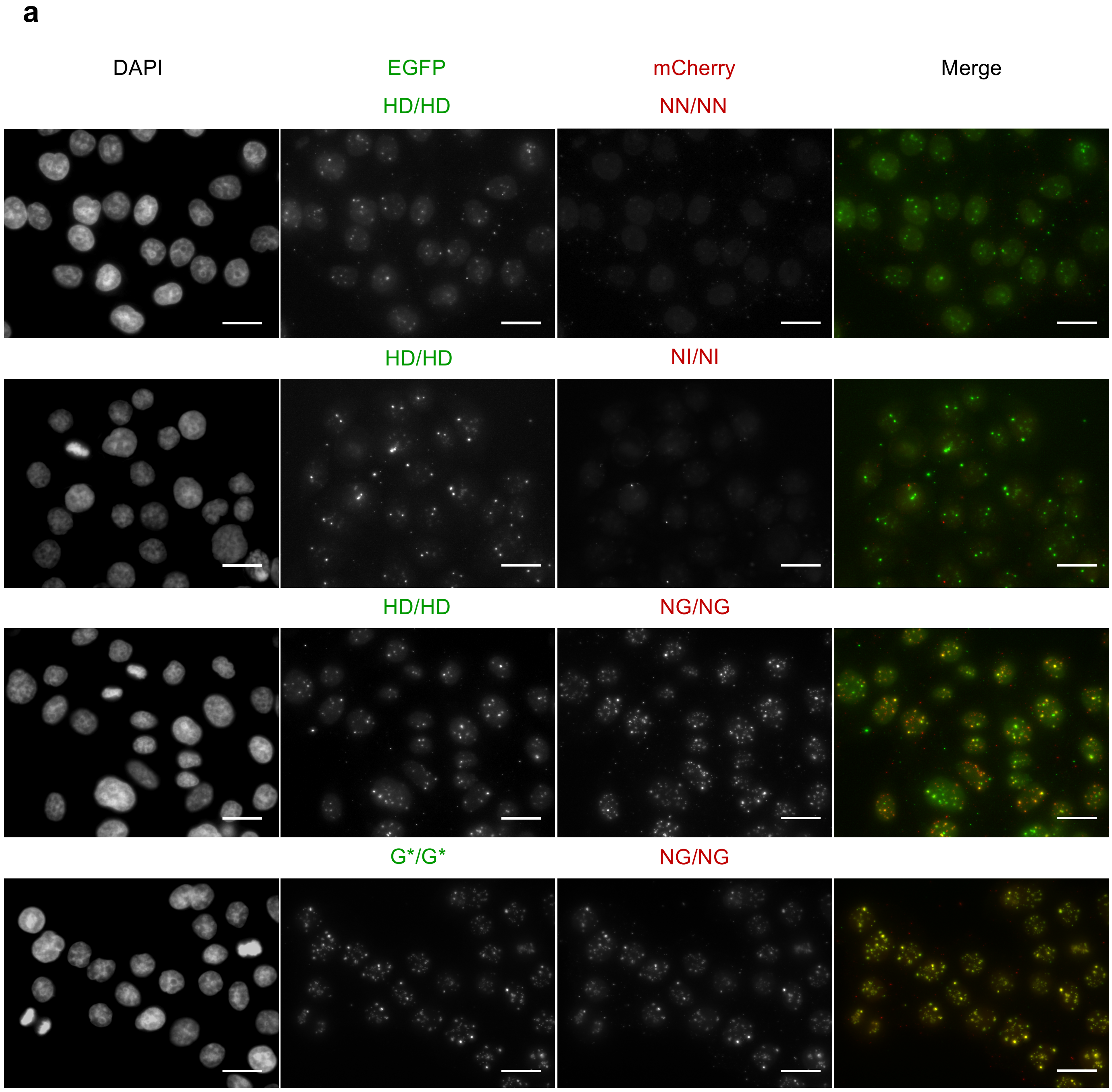

Figure S5. TALEs selectivity. a) HeLa co-stainings with TALE_2 bearing indicated repeats at positions 5 and 10. Scale bar is 20 μm.

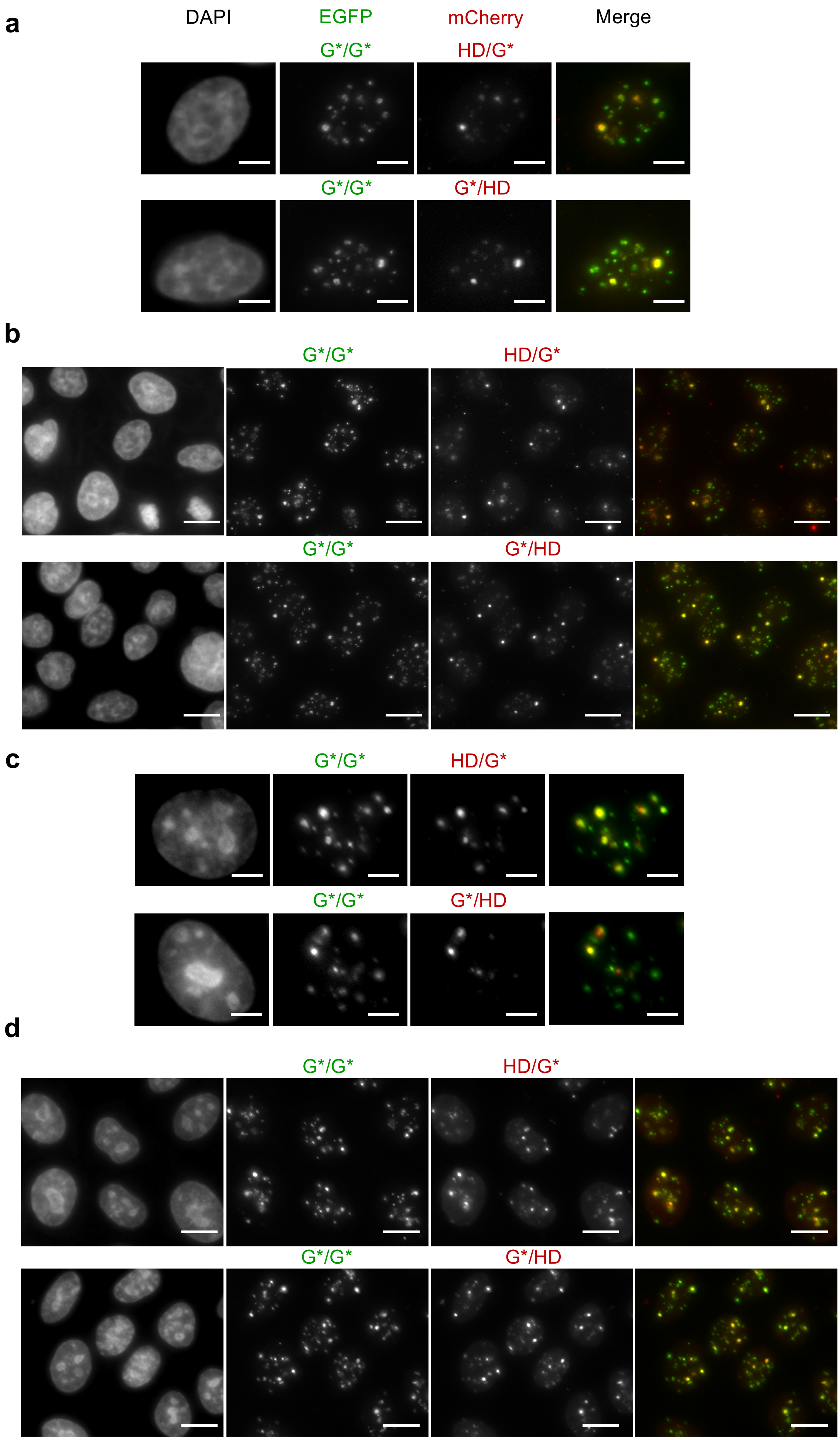
a
DAPI
EGFP
mCherry
Merge
G*/G*
HD/G*
G*/G*
G*/HD
b
G*/G*
HD/G*
G*/G*
G*/HD
c
G*/G*
HD/G*
G*/G*
G*/HD
d
G*/G*
HD/G*
G*/G*
G*/HD

Figure S6. TALEs selectivity in HeLa and HEK 293T cells. a) TALE stains with TALE_2 bearing indicated RVDs at positions 5 and 10 in HeLa cells. Scale bar is 5 μm **b)** TALE stain as in Fig. S6a showing multiple cells. Scale bar is 20 μm. **c)** TALE stains of HEK 293T cells with TALE_2 bearing indicated RVDs at positions 5 and 10. Scale bar is 5 μm. **d)** TALE stains as in Fig. S6c showing multiple cells. Scale bar is 20 μm.

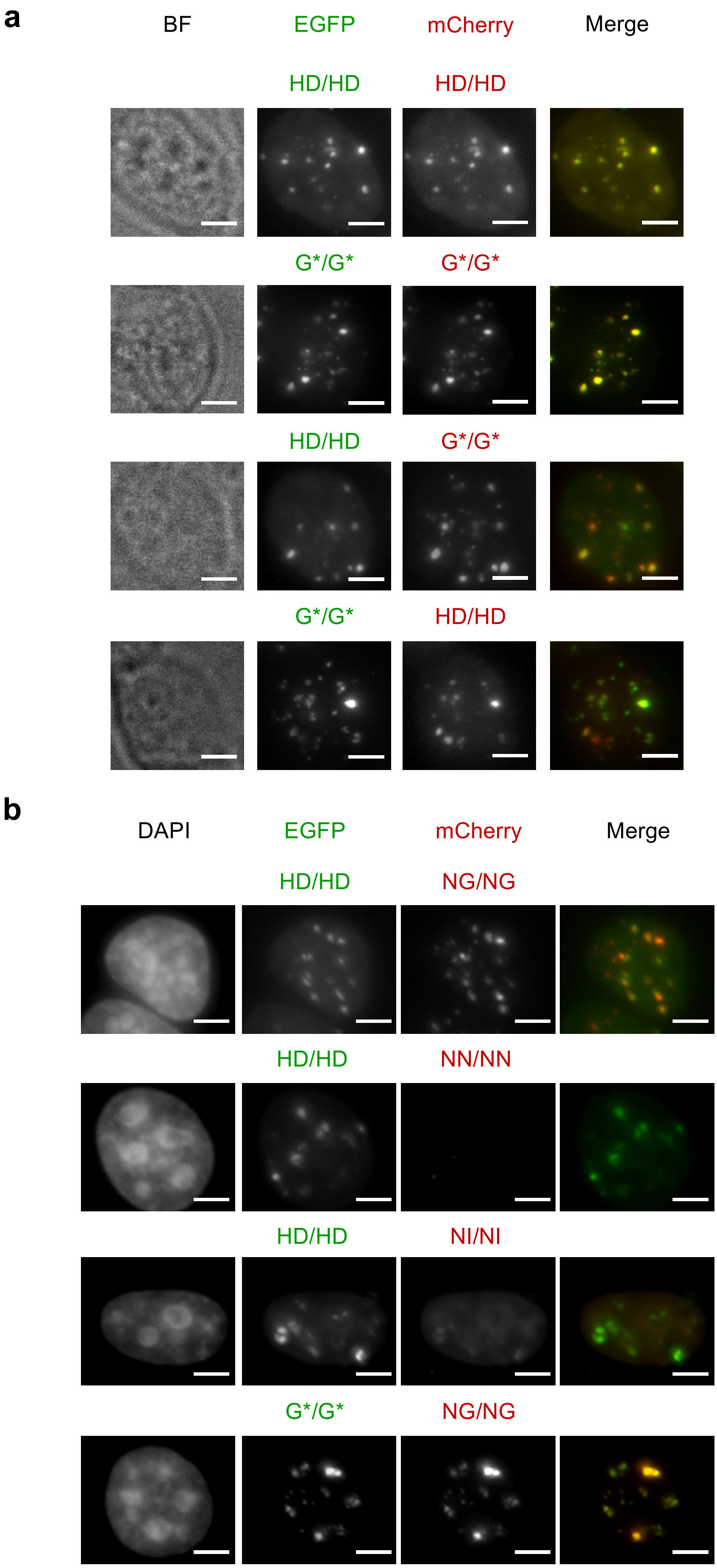
a
BF
EGFP
mCherry
Merge
HD/HD
HD/HD
G*/G*
G*/G*
HD/HD
G*/G*
G*/G*
HD/HD
b
DAPI
EGFP
mCherry
Merge
HD/HD
NG/NG
HD/HD
NN/NN
HD/HD
NI/NI
G*/G*
NG/NG

Figure S7. TALE selectivity in HEK 293T cells. a) TALE stains with TALE_2 bearing indicated RVDs at positions 5 and 10 in HEK 293T cells. Scale bar is 5 μm. **b)** Stains as in Fig. S7a with TALE_2 carrying the indicated RVDs. Scale bar is 5 μm.

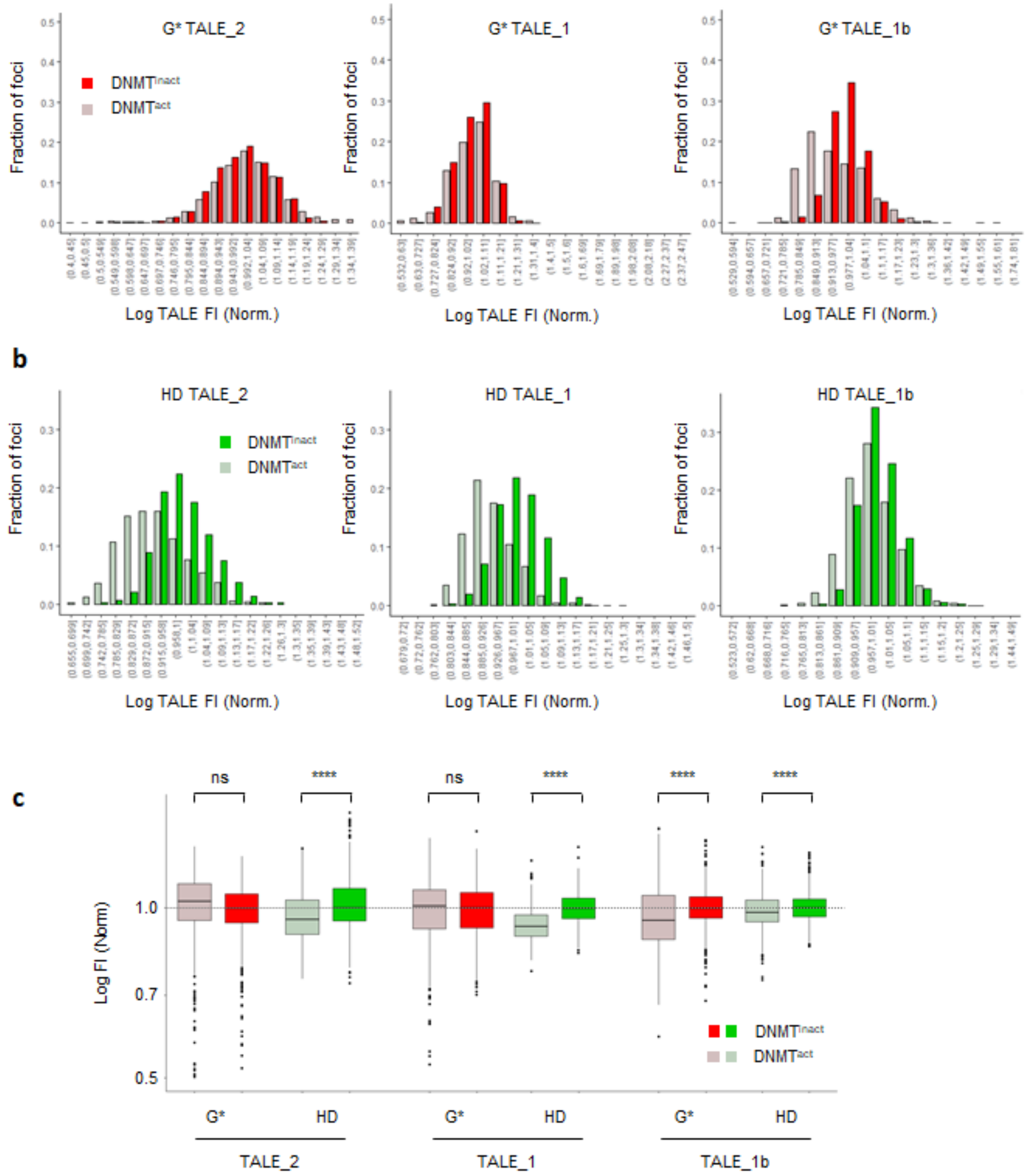

Figure S8. TALE staining and imaging of HEK 293T cells enables 5mC analysis at user-defined CpGs. Foci Analysis. a) Full histogram of G* TALE fluorescent intensities of foci (log transformed and normalized by the average fluorescent intensities of foci in DNMT^{inact} sample for each experiment) co-stained with G* and HD TALEs in cells transfected with either DNMT^{act} or DNMT^{inact}. **b)** Same as in Fig. S8a, but for HD TALEs fluorescent intensities. **c)** Box plots of data presented in Fig. S8a-b with t-test using as sample size the number of foci (N >1000 foci). See Data Analysis and Statistics section.

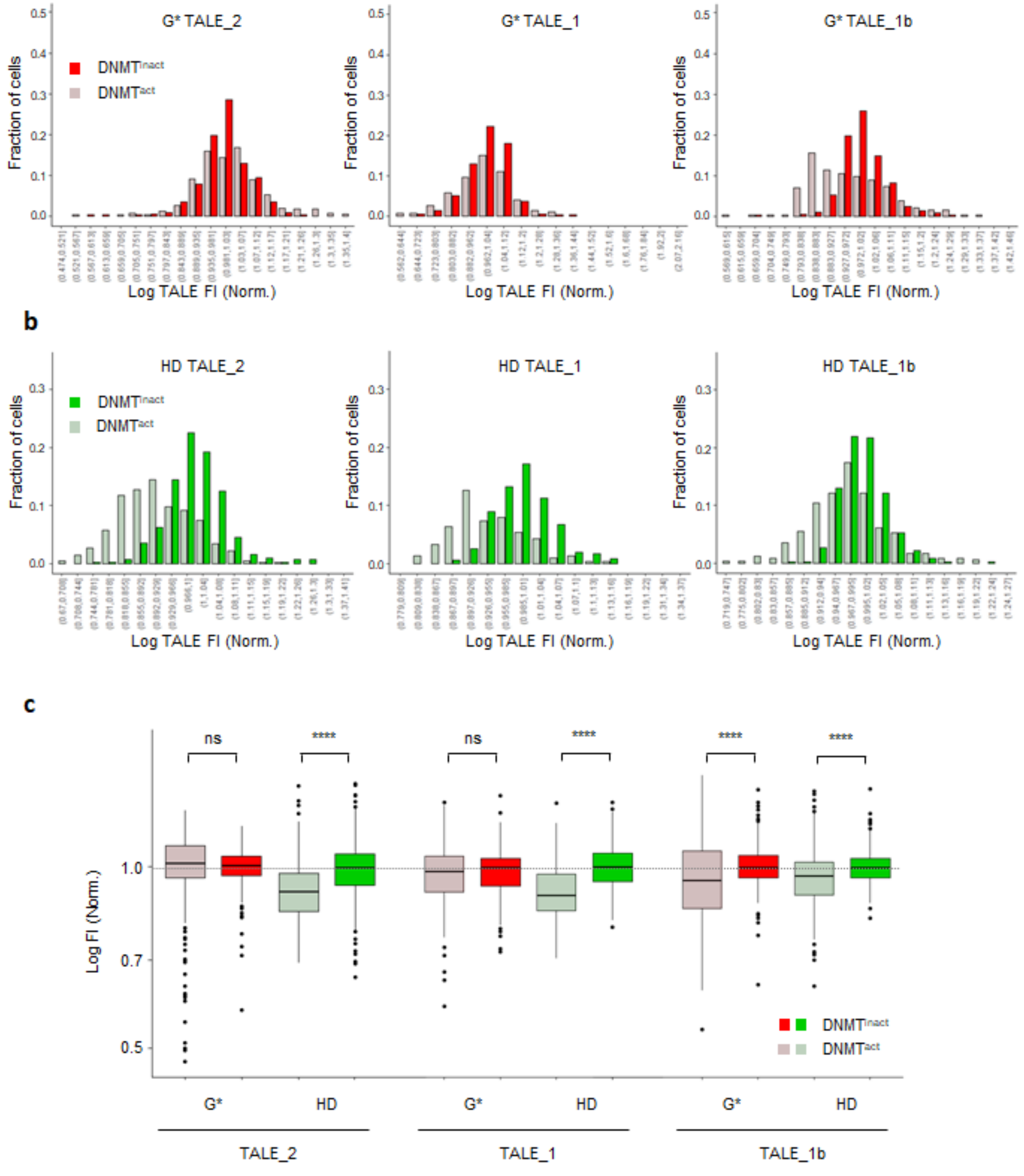

Figure S9. TALE staining and imaging of HEK 293T cells enables 5mC analysis at user-defined CpGs. Cell Analysis. a) Full histogram of G* TALE average fluorescent intensities of cells (log transformed and normalized by the average fluorescent intensities of foci within the same nucleus in DNMTinact sample for each experiment) co-stained with G* and HD TALEs in cells transfected with either DNMTact or DNMTinact. **b)** Same as in Fig. S8a, but for HD TALEs fluorescent intensities. **c)** Box plots of data presented in Fig. S8a-b with t-test using as sample size the number of cells (N > 200 cells). See Data Analysis and Statistics section.

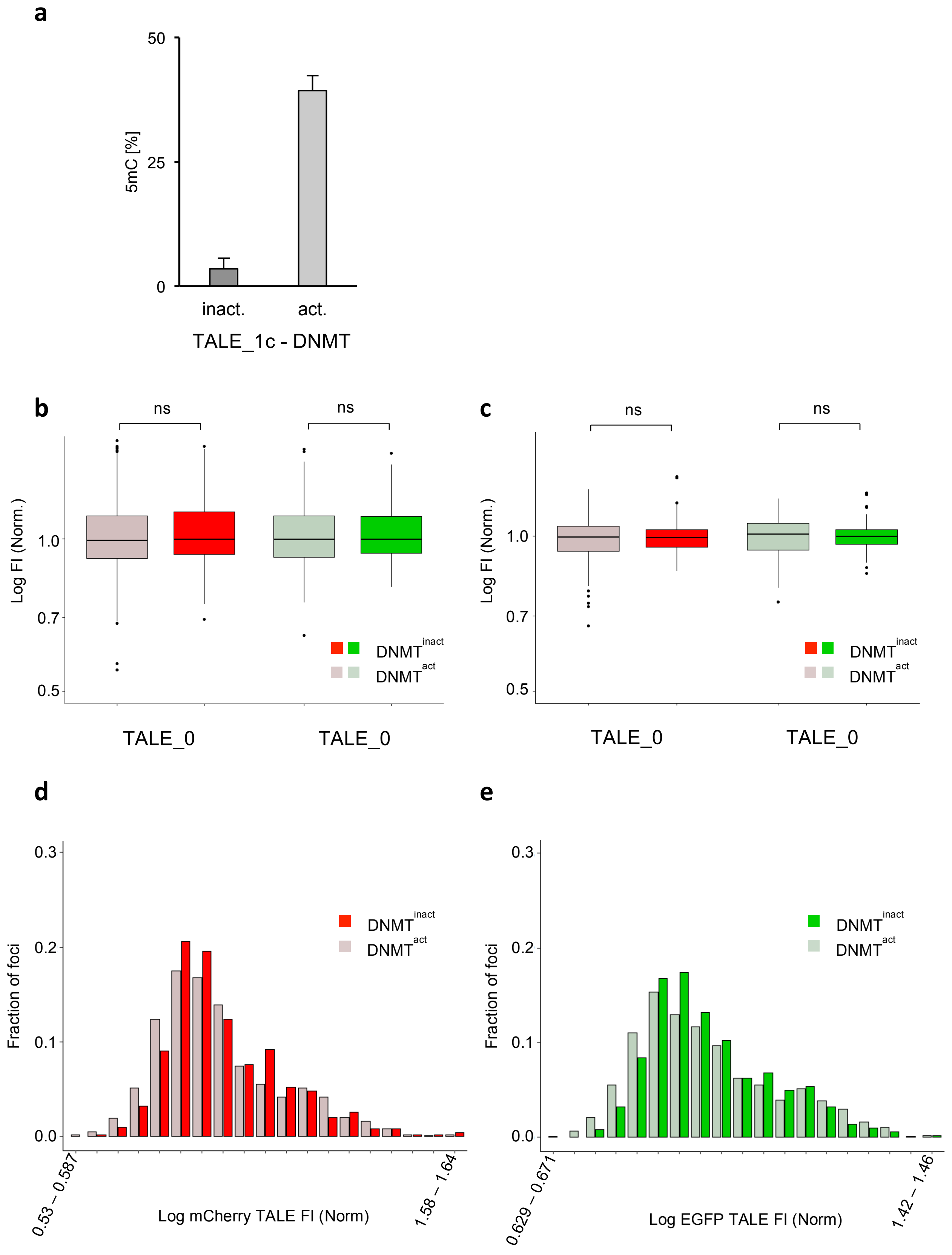
a
50
25
0
5mC [%]
inact.
act.
TALE_1c - DNMT
b
ns
ns
Log FI (Norm.)
1.0
0.7
0.5
DNMT inact
DNMT act
TALE_0
TALE_0
c
ns
ns
Log FI (Norm.)
1.0
0.7
0.5
DNMT inact
DNMT act
TALE_0
TALE_0
d
Fraction of foci
0.3
0.2
0.1
0.0
DNMT inact
DNMT act
0.53 – 0.587
1.58 – 1.64
Log mCherry TALE FI (Norm)
e
Fraction of foci
0.3
0.2
0.1
0.0
DNMT inact
DNMT act
0.629 – 0.671
1.42 – 1.46
Log EGFP TALE FI (Norm)

Figure S10. TALEs targeting CpG-free target sequence are not affected by 5mC. a) Pyrosequencing analysis of SATIII target CpG methylation for TALE_1c-DNMT^{act} or TALE_1c-DNMT^{inact} in HEK 293T cells. **b)** Log transformed fluorescent intensities of foci for mCherry (red) and EGFP TALE_0 (green) co-stains in DNMTact or DNMTinact (TALE_1c) transfected HEK 293T cells. Statistical analysis is a t-test using foci as sample size (N > 1000 foci). See Data Analysis and Statistics section. **c)** Boxplots as in Fig. S10b, but showing average fluorescent intensity of foci within each nucleus (log transformed and normalized), t-test was performed using number of cells as sample size (N > 200). See Data Analysis and Statistics section. **d)** Full histogram of mCherry TALE_0 fluorescent intensity of foci. **e)** Same as in Fig. S10e, but with EGFP TALE_0.

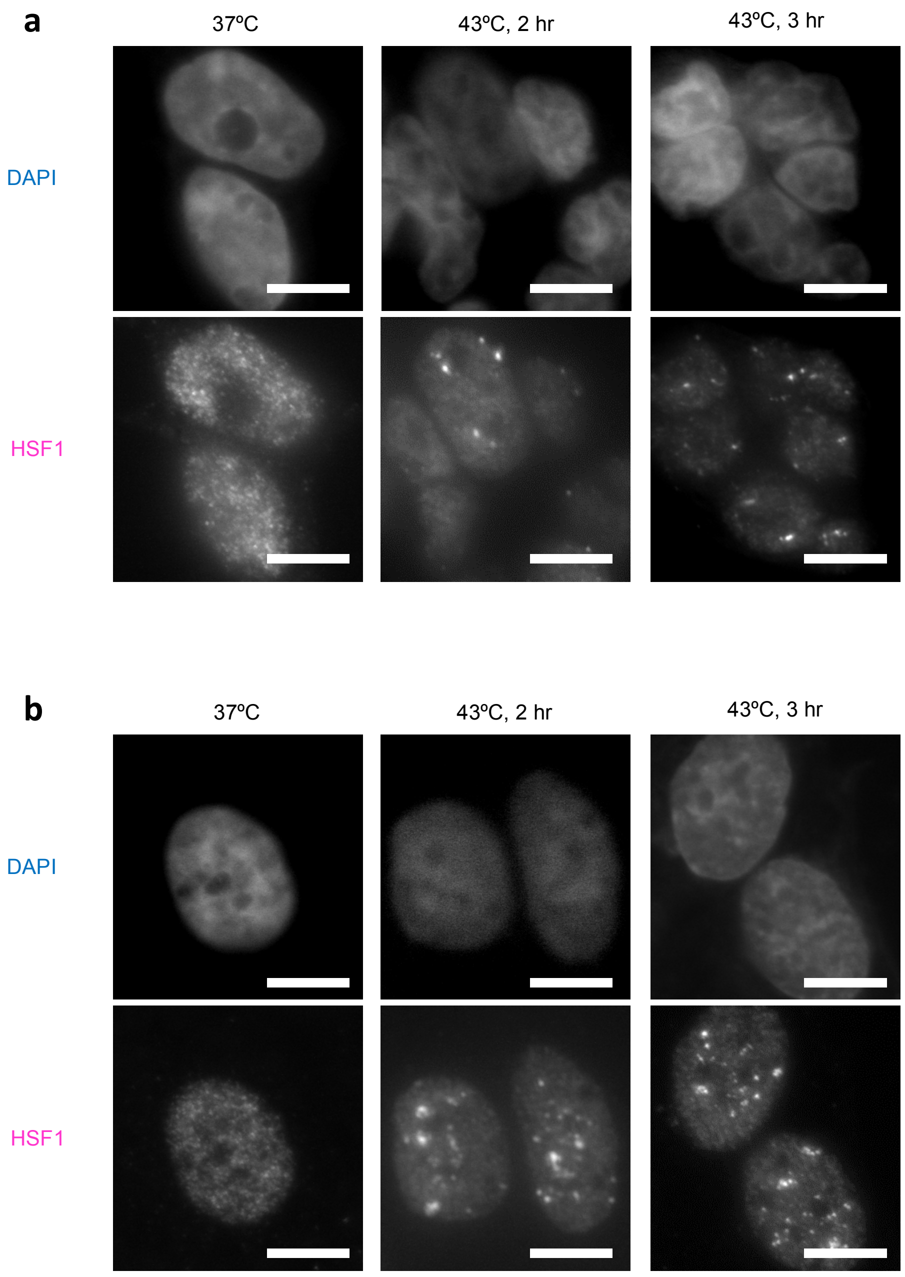

Figure S11. Nuclear stress body formation after Heat-Shock in HEK 293T and U2OS cells. a) Endogenous HSF1 immunostaining of HEK 293T cells grown at 37ºC or incubated at 43ºC for indicated times**.** Scale bar is 10 μm. **b)** HSF1 immunostaining as in Fig. S11a, but in U2OS cells. Scale bar is 10 μm.

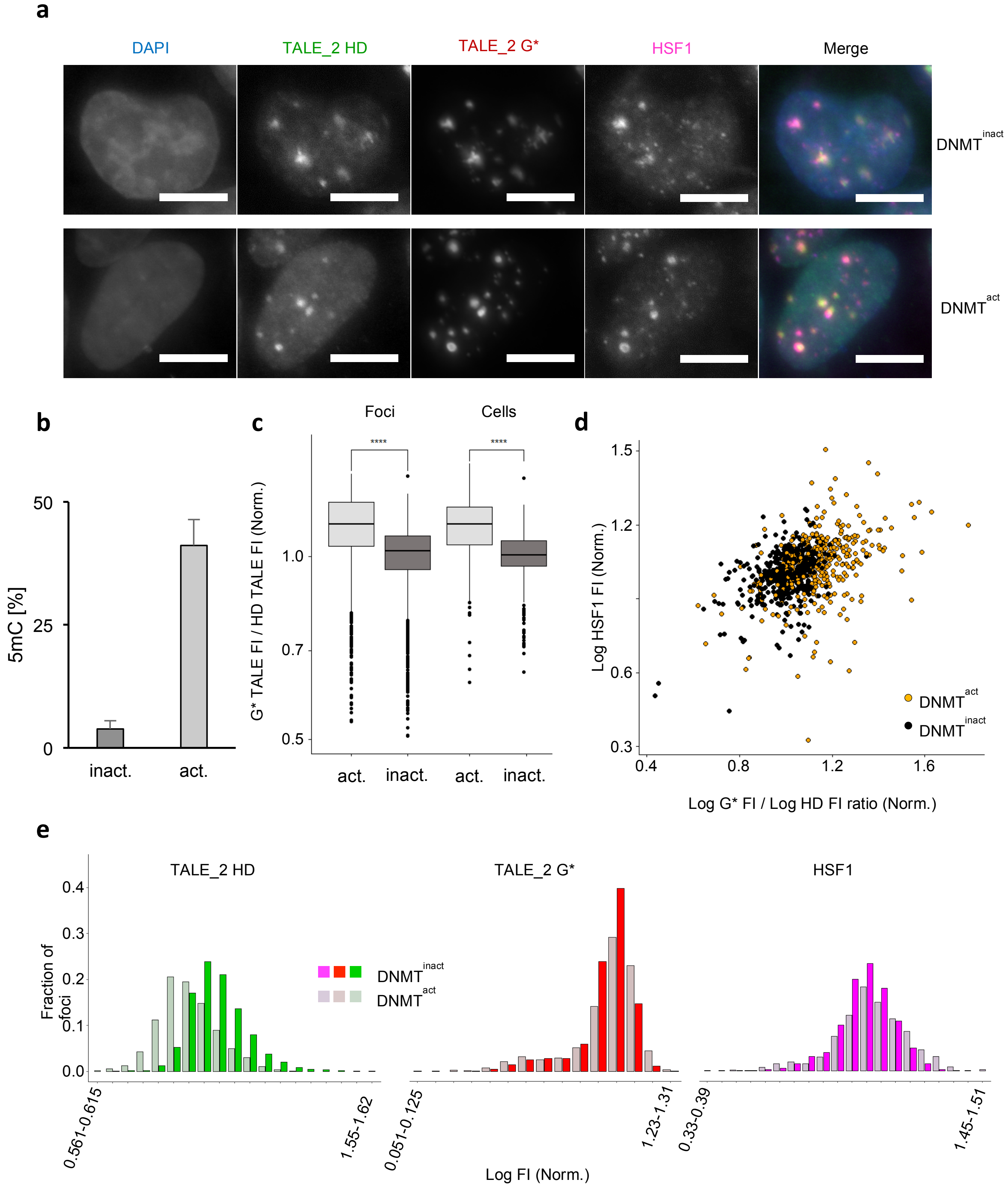
a
DAPI
TALE_2 HD
TALE_2 G*
HSF1
Merge
DNMT inact
DNMT act
b
5mC [%]
50
25
0
inact.
act.
c
Foci
Cells
****
****
G* TALE FI / HD TALE FI (Norm.)
1.0
0.7
0.5
act.
inact.
act.
inact.
d
Log HSF1 FI (Norm.)
1.5
1.2
0.6
0.3
0.4
0.8
1.2
1.6
DNMT act
DNMT inact
Log G* FI / Log HD FI ratio (Norm.)
e
TALE_2 HD
TALE_2 G*
HSF1
Fraction of foci
0.4
0.3
0.2
0.1
0.0
DNMT inact
DNMT act
0.561-0.615
1.55-1.62
0.051-0.125
1.23-1.31
0.33-0.39
1.45-1.51
Log FI (Norm.)

Figure S12. TALEs reveal a role of 5mC in the regulation of heat-shock-induced recruitment of HSF1 to SATIII U2OS cells. a) Co-stain of HD and G* TALE_2 combined with endogenous HSF1 immunostaining in U2OS cells. Cells were incubated for 2 hours at 43ºC. Scale bar is 10 μm. **b)** Pyrosequencing analysis of SATIII target CpG methylation for DNMT^{act} or DNMT^{inact} U2OS cells. **c)** Boxplot showing the ratio of G* TALE fluorescent intensity over HD TALE fluorescent intensity per foci and per cell(log transformed and normalized) in U2OS cells. T-test was perform using either the number of foci or the cells as sample size (N > 1000 foci, N > 200 cells). See Data Analysis and Statistics section. **d)** Scatter plot showing the ratio of G* fluorescent intensity over HD fluorescent intensity per cells versus fluorescent intensity of HSF1 (log transformed and normalized). **e)** Full histograms showing the fraction of foci with indicated fluorescent intensities for HD and G* TALEs and HSF1 immunostaining.

References

(1) Gieß, M.; Muñoz-López, Á.; Buchmuller, B.; Kubik, G.; Summerer, D. Programmable Protein-DNA Cross-Linking for the Direct Capture and Quantification of 5-Formylcytosine. *Journal of the American Chemical Society* **2019**, *141*, 9453–9457.

(2) Gibson, D. G.; Young, L.; Chuang, R.-Y.; Venter, J. C.; Hutchison, C. A.; Smith, H. O. Enzymatic assembly of DNA molecules up to several hundred kilobases. *Nature methods* **2009**, *6*, 343–345.

(3) Cermak, T.; Doyle, E. L.; Christian, M.; Wang, L.; Zhang, Y.; Schmidt, C.; Baller, J. A.; Somia, N. V.; Bogdanove, A. J.; Voytas, D. F. Efficient design and assembly of custom TALEN and other TAL effector-based constructs for DNA targeting. *Nucleic acids research* **2011**, *39*, e82.

(4) Shaner, N. C.; Campbell, R. E.; Steinbach, P. A.; Giepmans, B. N. G.; Palmer, A. E.; Tsien, R. Y. Improved monomeric red, orange and yellow fluorescent proteins derived from Discosoma sp. red fluorescent protein. *Nature biotechnology* **2004**, *22*, 1567–1572.

(5) Kubik, G.; Summerer, D. Achieving single-nucleotide resolution of 5-methylcytosine detection with TALEs. *Chembiochem : a European journal of chemical biology* **2015**, *16*, 228–231.

(6) Rathi, P.; Witte, A.; Summerer, D. Engineering DNA Backbone Interactions Results in TALE Scaffolds with Enhanced 5-Methylcytosine Selectivity. *Scientific reports* **2017**, *7*, 15067.

(7) Schindelin, J.; Arganda-Carreras, I.; Frise, E.; Kaynig, V.; Longair, M.; Pietzsch, T.; Preibisch, S.; Rueden, C.; Saalfeld, S.; Schmid, B. *et al*. Fiji - an Open Source platform for biological image analysis. *Nature methods* **2012**, *9*.

(8) Schneider, C. A.; Rasband, W. S.; Eliceiri, K. W. NIH Image to ImageJ: 25 years of Image Analysis. *Nature methods* **2012**, *9*, 671–675.

(9) Haub, P.; Meckel, T. GaussFit OnSpot **2014**.

(10) Edelstein, A. D.; Tsuchida, M. A.; Amodaj, N.; Pinkard, H.; Vale, R. D.; Stuurman, N. Advanced methods of microscope control using μManager software. *Journal of biological methods*, *1*.

(11) Dowle, M.; Srinivasan, A. data.table: Extension of `data.frame`. R package version 1.12.6. https://cran.r-project.org/package=data.table.

(12) R Core Team. R: A language and environment for statistical computing. https://www.r-project.org/.

(13) H. Wickham. *ggplot2: Elegant Graphics for Data Analysis*; Springer-Verlag: New York, 2016.

(14) Kassambara, A. ggpubr: 'ggplot2' Based Publication Ready Plots. R package version 0.2.4. https://cran.r-project.org/package=ggpubr.

Appendix 1 – Entry Vector Maps

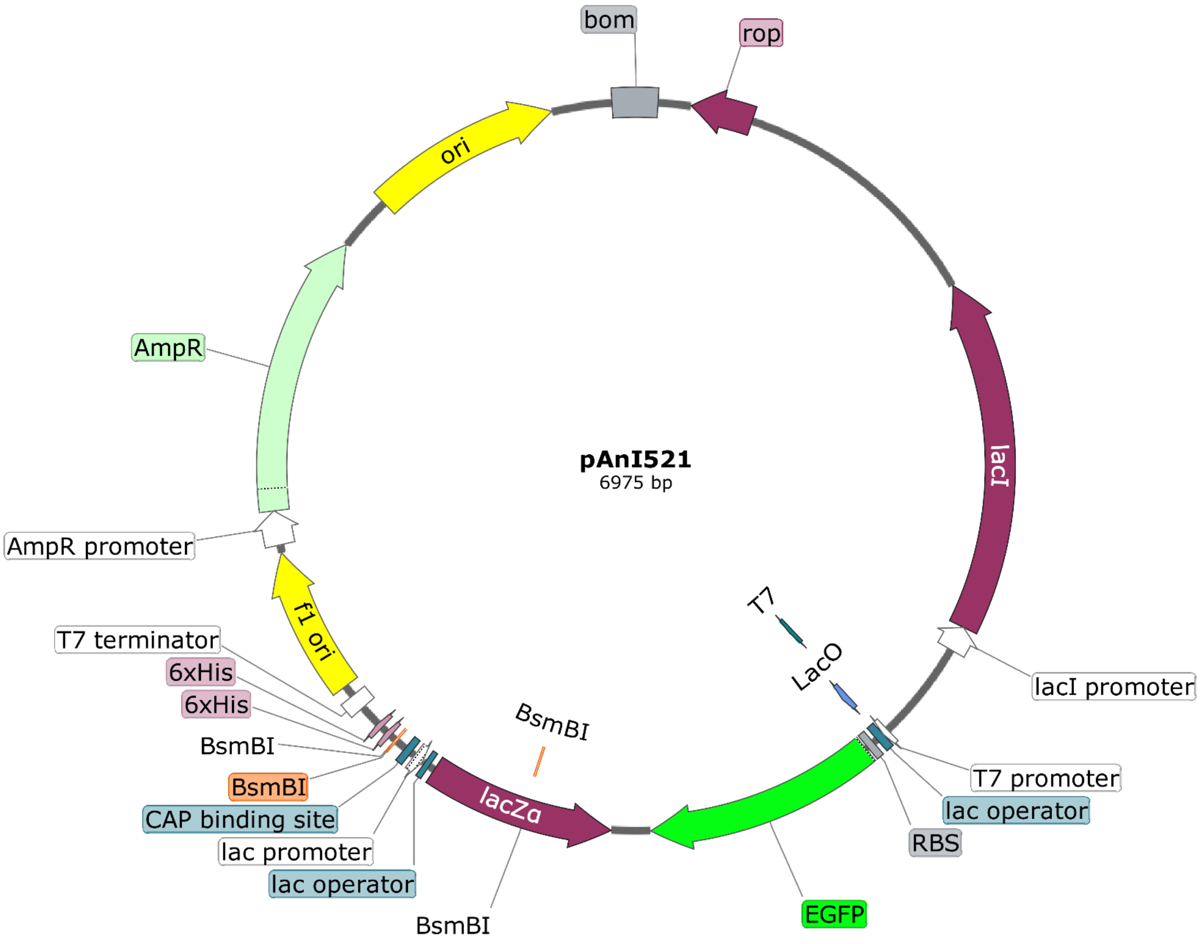

pAnI521

GG2 Entry Vector for bacterial expression of TALEs fused to N-terminal EGFP and C-terminal 6xHis for protein purification.

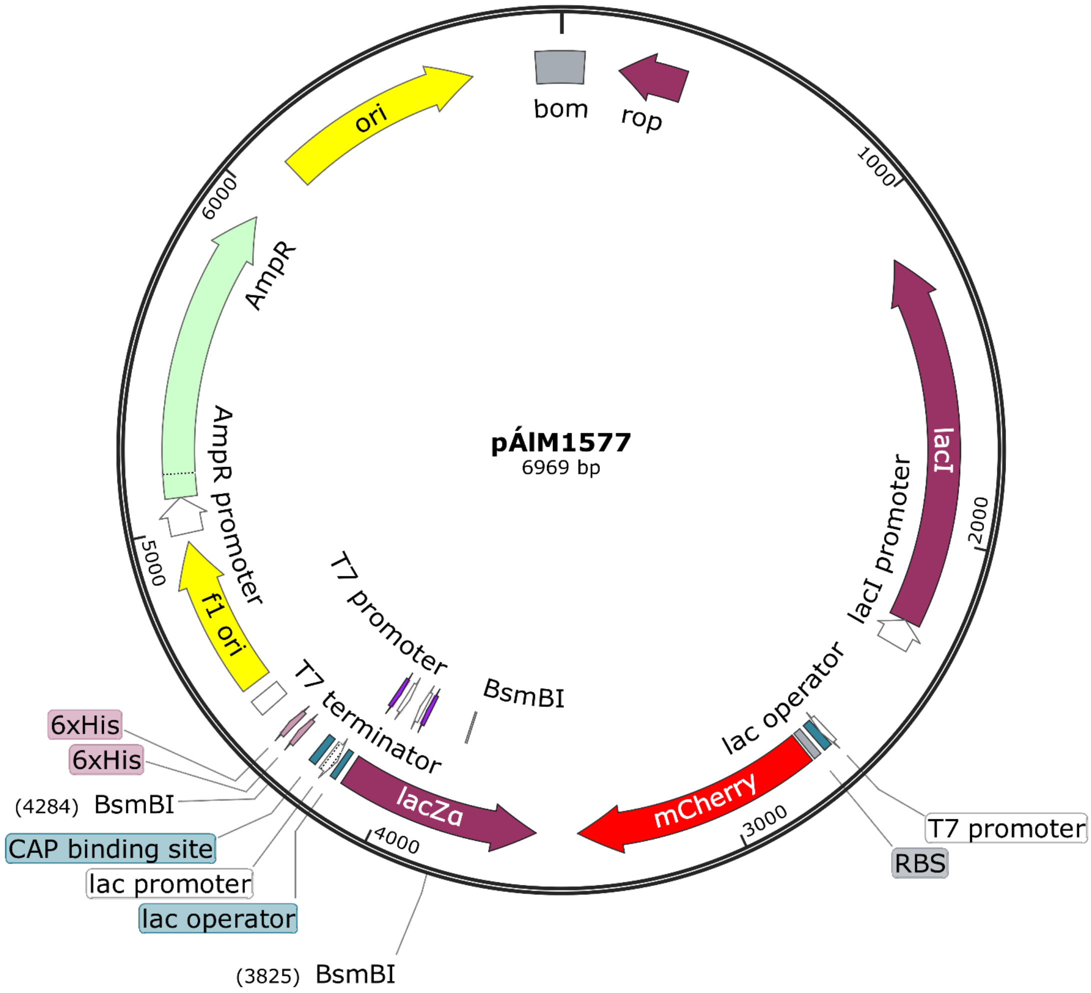

pÁlM1577

Vector for bacterial expression of TALEs fused to N-terminal mCherry and C-terminal 6xHis for protein purification.

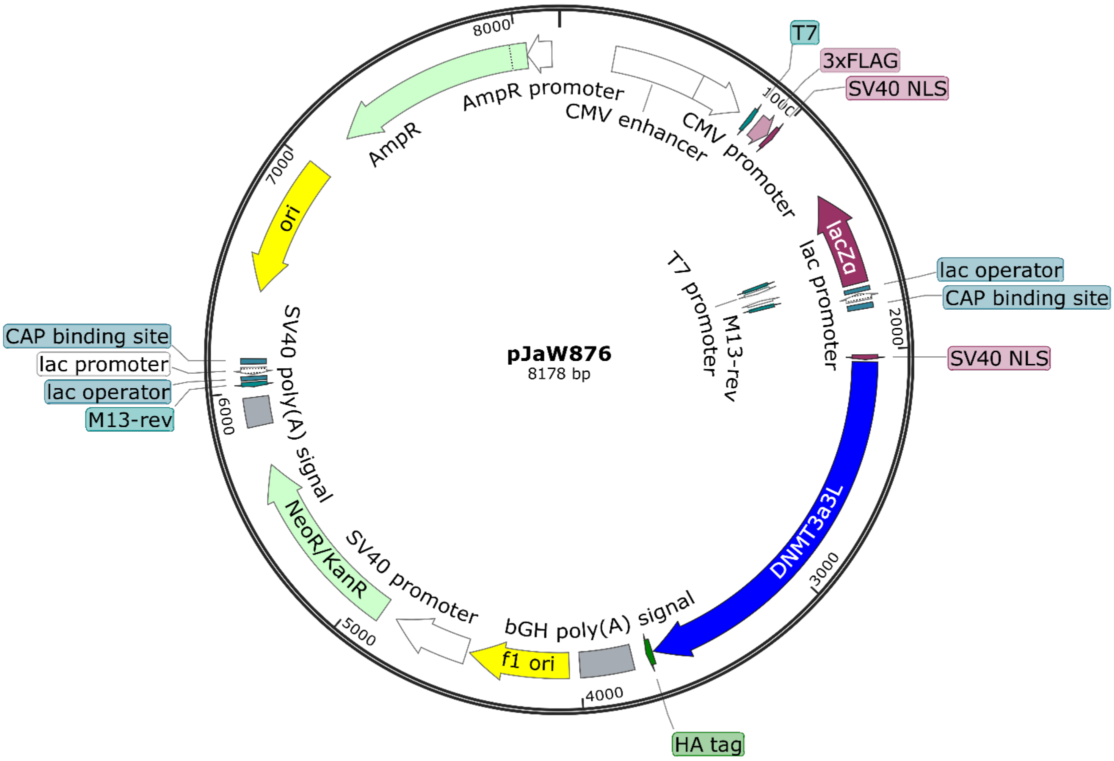

pJaW876

Vector for mammalian expression of TALEs fused to C-terminal DNMT3a3L and HA-tag and N-terminal 3XFLAG-tag for for live-cell site-specific DNA methylation.

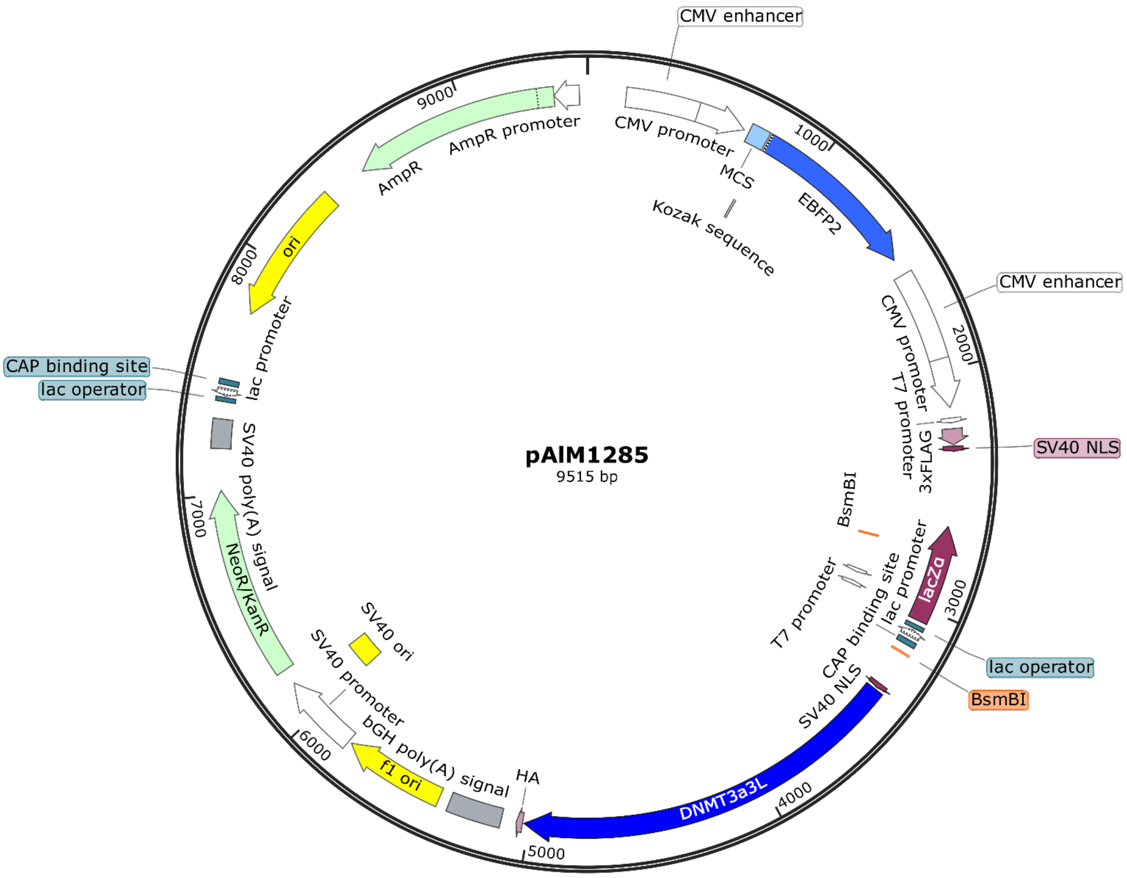

pÁlM1285

Vector for mammalian expression of TALEs fused to C-terminal DNMT3a3L and HA-tag and N-terminal 3XFLAG-tag for for live-cell site-specific DNA methylation. It also contains an EBFP2 fluorophore under the same promoter (CMV) as transfection control.

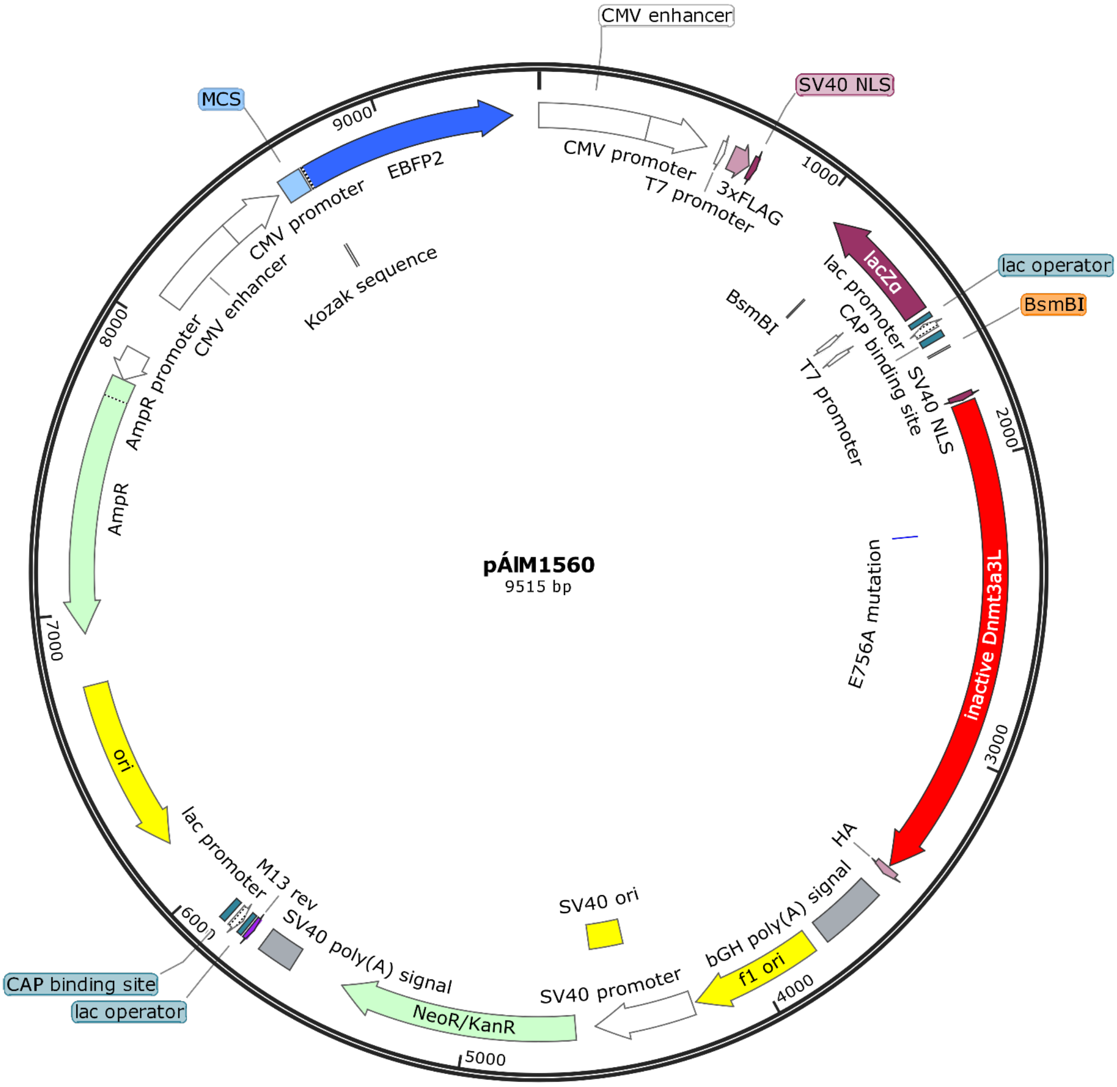

pÁIM1560

Vector for mammalian expression of TALEs fused to C-terminal inactive DNMT3a3L (carrying E756A mutation) and HA-tag and N-terminal 3XFLAG-tag. It also contains an EBFP2 fluorophore under the same promoter (CMV) as transfection control. This construct is used as negative control for site-specific DNA-methylation.
